# Using the Community Perception Tracker (CPT) to inform COVID-19 response in Lebanon and Zimbabwe: a qualitative methods evaluation

**DOI:** 10.1186/s12889-025-23755-4

**Published:** 2025-08-19

**Authors:** Fiona Majorin, Anika Jain, Christine Haddad, Eddington Zinyandu, Ghassan Gharzeddine, Mutsawashe Chitando, Aline Maalouf, Ntandoyenkosi Sithole, Rita Doumit, Raissa Azzalini, Thomas Heath, Janet Seeley, Sian White

**Affiliations:** 1 Department of Disease Control, London School of Hygiene and Tropical Medicine, Keppel Street, London, UK; 2 Independent Consultant, New York, USA; 3 Oxfam Lebanon, Beirut, Lebanon; 4 Action Against Hunger Zimbabwe, Harare, Zimbabwe; 5 Alice Ramez Chagoury School of Nursing, Lebanese American University, Beirut, Lebanon; 6 Oxfam, John Smith Drive, Oxford, UK; 7 Action Contre La Faim, Boulevard Douaumont, Paris, France; 8 Department of Global Health & Development, London School of Hygiene and Tropical Medicine, Keppel Street, London, UK

**Keywords:** Community engagement, COVID-19, Process evaluation, Zimbabwe, Lebanon, Perceptions

## Abstract

**Background:**

Despite the recognized importance of community engagement during disease outbreaks, methods describing how to operationalise engagement are lacking. The Community Perception Tracker (CPT) was designed by Oxfam to systematically record real-time information on disease perceptions and outbreak response actions in order to adapt programmes.

**Methods:**

We conducted a phased, qualitative methods, process evaluation in Zimbabwe and Lebanon to understand whether the CPT approach was a feasible way to incorporate community perceptions into COVID-19 response programming and whether this resulted in more relevant programming. We conducted 3 rounds of interviews with 15 staff using the CPT, analysed programmatic data, and conducted multiple rounds of phone-based interviews with outbreak-affected populations (41 to 50 participants per country each round). Qualitative data were thematically analysed and quantitative data descriptively summarized.

**Results:**

Initially CPT implementing staff struggled to differentiate how the CPT differed from other monitoring tools that they were familiar with and felt that the training did not convey the full process and its value. However, with practise, collaboration and iterative improvements to the recommended CPT steps, staff found the process to be feasible and a significant value-add to their programming. Staff initially focused more on quantitively summarizing perceptions but eventually developed processes for maximizing the qualitative data on perceptions too. Trends emerging from the CPT led to frequent programmatic tweaks to COVID-19 messaging and product distributions. Emergent trends in perceptions also led staff to work cross-sectorally and advocate to other actors on behalf of populations. Outbreak-affected populations exposed to the programmes reported high levels of knowledge about COVID-19 and reported they practiced preventative behaviours, although this waned with time. Most population members also felt the COVID-19 programmes were relevant to their needs and said that non-government organisations were a trusted source of information.

**Conclusions:**

The CPT appears to be a promising approach for ensuring that community engagement is undertaken systematically and that community perspectives are actively incorporated to improve programming. While crisis-affected populations generally found the programmes to be useful and relevant and to have influenced their knowledge and behaviours, it is not possible to attribute this to the CPT approach due to the study design.

**Supplementary Information:**

The online version contains supplementary material available at 10.1186/s12889-025-23755-4.

## Background

The COVID-19 pandemic generated widespread fear, misinformation, and mistrust in public health measures [1–3]. Along with the vaccine, preventative behaviours (like hand hygiene, mask use, and physical distancing) have been important in controlling the spread of infection [4–6]. To facilitate adoption of preventative behaviours it is critical to understand community perceptions around a disease like COVID-19 and how these change over time. However, mechanisms for recording and sharing accurate and timely data on people’s perceptions during outbreaks have historically been weak or lacking [7–10], particularly in lower-and middle-income countries (LMICs) where analysis of social media trends may not reflect population views [11, 12]. Without these data, response programmes cannot always be adapted to people’s preferences, needs and knowledge and this in turn may have a detrimental impact on programme effectiveness.

**Fig. 1:**
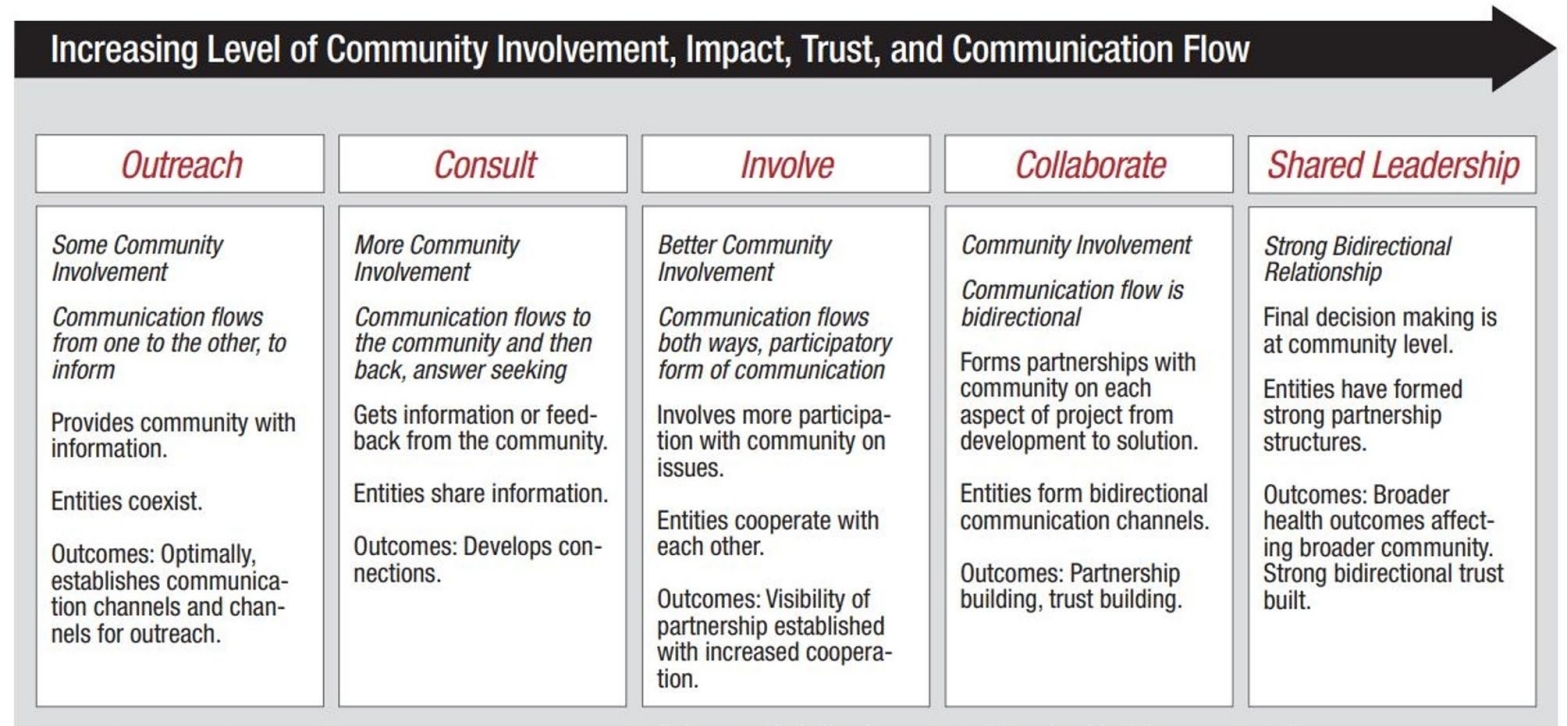
The Community Engagement Continuum (18)

Community engagement in health promotion is a planned and dynamic process of developing relationships that enable members of communities and public health professionals to work together to address health-related and wellbeing-related issues [13]. The Sphere standards definition adds that the process is done so that crisis-affected people have more control over humanitarian responses and its impact on them [14]. In practice this means that outbreak response actors such as government or non-government actors, must work with affected populations in order to design programmes relevant to their needs and priorities. This process requires engaging with local realities to understand barriers to preventative behaviours, and what is needed to facilitate people’s decision-making capacities in order to stop disease transmission [15, 16]. Community Engagement can be thought of as a continuum, of community involvement as outlined in the Community Engagement Continuum[17, 18] (Fig. 1). The continuum proposes criteria for assessing the depth of community engagement within programming. At the lowest level are programmes which just manage to achieve ‘outreach’, but with increased ‘community involvement, impact, trust and communication’, programmes can move up the continuum and ultimately towards ‘shared leadership’ of programming. Community engagement was identified as a key pillar of the World Health Organisation’s COVID-19 Strategic Preparedness and Response Plan [6] and has been shown to be particularly important in prior disease outbreaks [19].

During prior outbreaks of cholera, Ebola and Zika, it has been suggested that community engagement processes helped implementing organisations to build trust with communities, communicate effectively, identify local solutions, fill gaps in humanitarian response, and allowed for programmes to be iteratively adapted to meet emerging needs [19–23]. However, outbreak responses are typically designed rapidly and implementing organisations rely heavily on epidemiological data to inform policies and public health recommendations [7]. Additionally, community engagement initiatives may fail to understand social dynamics and local social, economic and political interests which influence the way programmes are designed and implemented [16, 24]. As such programmes can easily be designed and delivered in a top-down manner resulting in response programmes that lack contextual adaptation, are viewed by different members of a population as not being relevant to their needs, and are sometimes met with resistance from community members [25]. Understandably, such programmes are more likely to fail to achieve their desired aims or contribute to sustainable behaviour change [26, 27].

Despite community engagement being an increasing focus in academic publications, strategic guidelines and humanitarian standards, and being recognised by donors as a necessary component of project design and implementation, the practicalities of how to engage communities effectively are often poorly described. Reviews of community engagement and summaries of lessons learned from outbreak responses, have found the following elements as effective in outbreak responses: establishing mechanisms for a two-way dialogue between community members and response teams; having an understanding of the context in which a programme is taking place, including the complexity of local social dynamics; developing partnerships; understanding how knowledge, perceptions and behaviours develop over time;

and creating feedback loops so that programming is adapted based on lessons learned [7, 16, 20, 21, 28]. However, the unprecedented scale of the COVID-19 pandemic, and the need to reduce in-person interactions made applying these principles particularly challenging.

### Community perception tracker

Oxfam’s Community Perception Tracker (CPT) was developed and piloted during the 2018–19 Ebola outbreak in Democratic Republic of Congo (DRC). The CPT approach encourages programme implementation staff and partners to actively listen to communities throughout all aspects of their response work and iteratively improve programming based on findings and trends in perceptions. The CPT implementation process is intended to follow 6 steps [29]. 1) Data collection: where implementation staff listen and capture community members’ perceptions about the outbreak, whilst conducting programmatic activities. This is not done as a purposive data collection exercise with community members, but rather occurs during routine interactions with community members. 2) Data analysis: where data is cleaned, validated and analysed. 3) Regular meetings and discussions based on the findings of the analysis: staff and partners discuss potential recommendations/actions. 4) Triangulation with other actors and/or other teams within the organisation. 5) Adapting activities and influencing others: the programme is adapted or advocacy and influencing other actors to facilitate change in areas where they can’t respond. 6) Follow up activities and monitoring. A more detailed summary of the CPT process is provided in Supplementary Material 1 following the Template for Intervention Description and Replication (TIDieR) checklist [30].

When the COVID-19 pandemic started, Oxfam and its partners decided to scale-up the approach across 12 countries. In this manuscript, we describe a process evaluation of the CPT as used by Oxfam and partners in Lebanon, and Action Against Hunger and partners in Zimbabwe as part of their COVID-19 response. These organisations and their partners are referred to as the ‘implementation partners’ in this manuscript. This process evaluation aimed to build an understanding of whether the CPT approach was a feasible way of incorporating community engagement into COVID-19 response programming and whether its inclusion meant that response programmes were seen as relevant and acceptable by members of affected populations.

## Methods

### Study settings

**Fig. 2:**
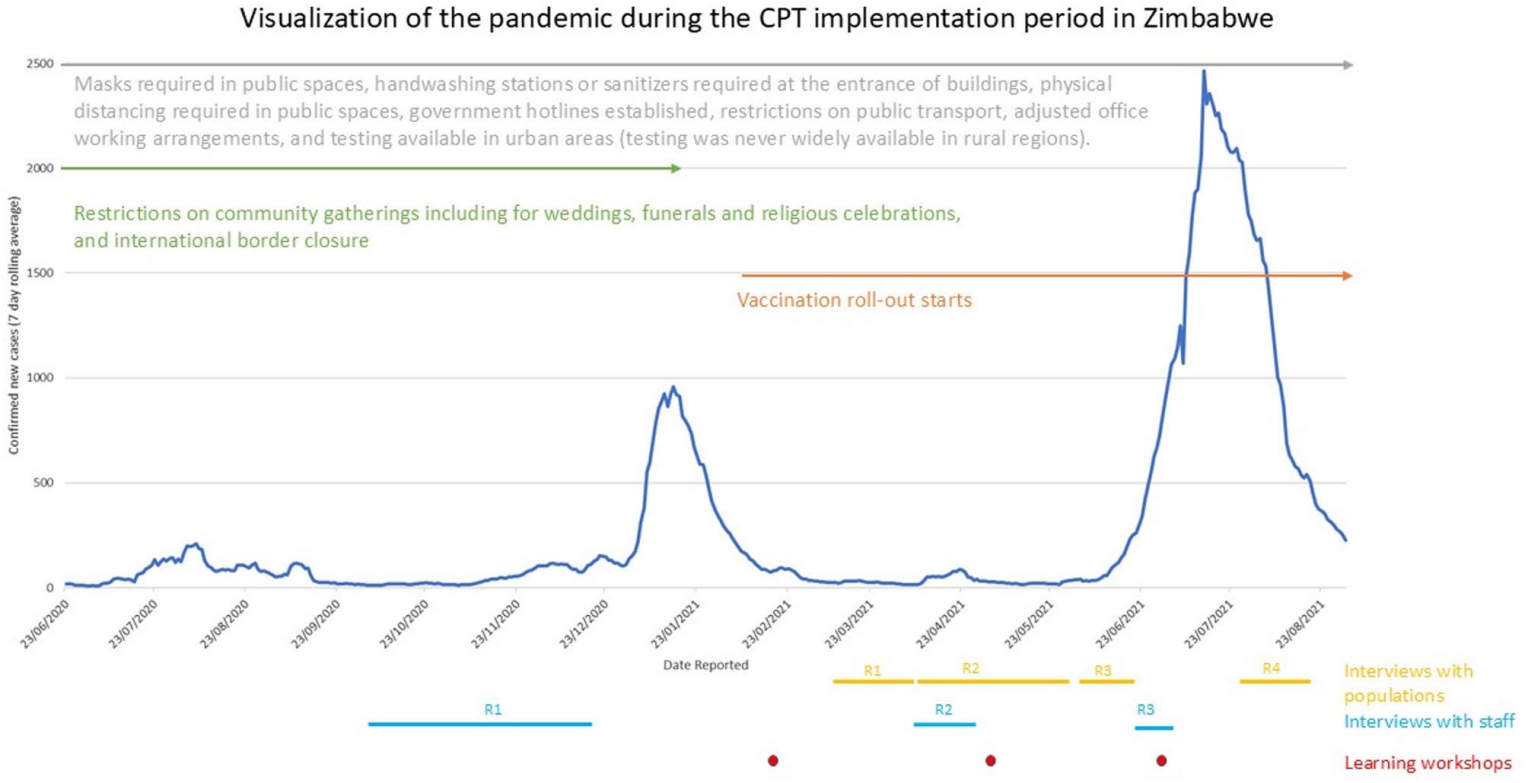
Visualization of the pandemic during the CPT implementation period in Zimbabwe including the epidemic curve [31], national COVID-19 policy milestones and the periods of data collection for this study

**Fig. 3:**
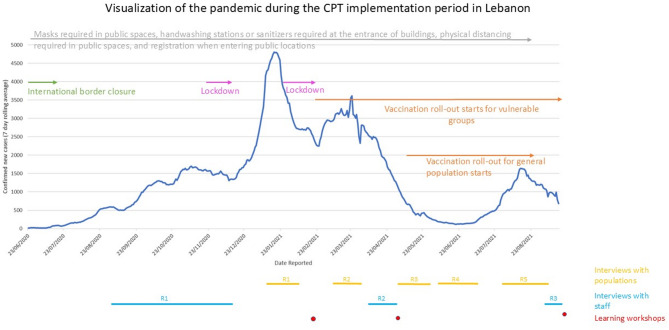
Visualization of the pandemic in Lebanon during the CPT implementation period including the Epidemic curve [31], national COVID-19 policy milestones and the periods of data collection for this study

The study was carried out in drought-affected regions of Zimbabwe and informal settlements for Syrian refugees in Lebanon. Thus, communities in both sites were dealing with the consequences of an existing crisis as well as the COVID-19 pandemic. Both countries experienced several waves of COVID-19 cases and national lockdown and prevention policies to control the spread of cases. Figures 2 and 3 show the epidemic curve in both countries, key policy milestones and the periods of data collection for this study.

In Zimbabwe, the study took place in Mwenezi and Chiredzi, in Masvingo province, both arid regions that are drought prone. The implementation partners in Zimbabwe were Action Against Hunger, Africa AHEAD and Nutrition Action Zimbabwe (NAZ). In these communities, the organisations were involved in hygiene promotion, sharing information on COVID-19, assisting in the development of community action plans, helping to provide handwashing stations and soap, waterpoint construction or rehabilitation, and conducting trainings on infection prevention for key stakeholders like community health workers and local leaders. WASH work was complemented by a large nutrition and food security programme involving cash distributions, distribution of products to improve livelihoods (chickens, tools, etc.) and associated training.

In Lebanon, the project locations were Saaide and Bouday in North Bekaa near the Syrian border. The implementation partners in Lebanon were Oxfam and Nabad for Development, who have been operating in 93 settlements in this area since 2013, providing water, sanitation, and hygiene (WASH) and protection programming. The informal settlements were considered high risk during the pandemic because they were set up as temporary, informal tented settlements and tend to be overcrowded. During COVID-19, Oxfam and Nabad’s programming focused on improving WASH standards within the informal settlements, improving refugee access to reliable and comprehensive information about COVID-19, and encouraging COVID-19 prevention behaviours as per national guidelines. The main activities of their programme included distributing hygiene kits, ensuring adequate water quality and quantities, ensuring functionality of existing WASH facilities, establishing WhatsApp groups with trusted focal people within the camps, and managing a hotline which people could call to ask questions.

### Study design

**Fig. 4:**
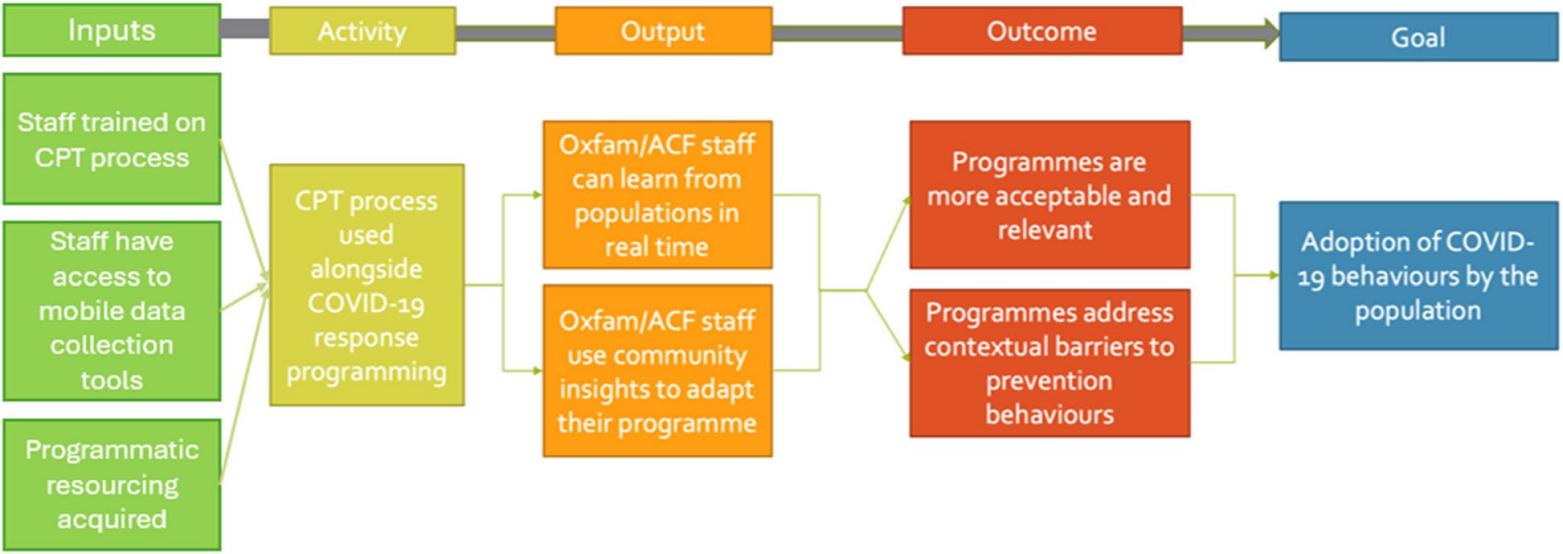
Hypothesized theory of change used to inform the study design

We adopted a phased process evaluation to assess the CPT process across a hypothesised theory of change (Fig. 4). The CPT theory of change was developed by the research team prior to the evaluation, following discussions with the Oxfam staff who had developed the approach. The hypothesis was that the CPT would provide humanitarian organisations with a mode of learning from outbreak-affected populations in real time. These community-generated insights should assist humanitarians to adapt and improve their programming to become more acceptable and relevant, and to address key behavioural barriers. According to this theory of change, programmes informed by CPT would therefore be more likely to affect COVID-19 prevention behaviours.

The approach was ‘phased’ because we recognised that the nature of the pandemic was so unprecedented that findings from this research could be of immediate relevance to the CPT implementation teams. Therefore, learning workshops were held allowing research staff to share preliminary findings with implementation teams. Findings led to a consensus on strategies to improve the CPT which were then implemented and monitored over the remaining study period.

The study used a mix of methods including qualitative interviews with staff, structured phone-based interviews with affected populations and an analysis of programmatic data. Observational methods for assessing community-level behaviour were originally planned but dropped due to feasibility and safety concerns. The study methods and their objectives are described in Table 1.

**Table 1:** Summary of research methods

Objectives	Methods	Number of participants	Data collection period and timeline
Understand whether the CPT enables staff within humanitarian organisations to learn from populations in real time and use community insights to adapt their programming	Reports and learning workshops documenting programmatic changes	N/A	Reports as developed during routine programming, 3 learning workshops
	In-depth qualitative interviews with CPT staff	Round 1: 15 staff (7 in Lebanon, 8 in Zimbabwe) Round 2: 14 staff (7 in Lebanon, 7 in Zimbabwe) Round 3: 14 staff (7 in Lebanon, 7 in Zimbabwe)	3 time points over the course of CPT implementation (at the beginning of the CPT project, mid-way through and at the end)
Understand whether programmatic adjustments made by humanitarians based on CPT insights made them more acceptable and relevant, addressed contextual barriers to prevention behaviours and ultimately led to behavioural change	Phone-based interviews with populations	Round 1: 100 participants (50 in Lebanon, 50 in Zimbabwe) Round 2: 97 participants 47 in Lebanon, 50 in Zimbabwe) Round 3: 97 participants (47 in Lebanon, 50 in Zimbabwe) Round 4: 87 participants (46 in Lebanon, 41 in Zimbabwe) Round 5: 45 participants from Lebanon*	4 time points in Zimbabwe and 5 time points in Lebanon over the course of CPT implementation period

^*^ Supplementary Material 2 includes flow charts which describes participants lost to follow up and subsequent recruitment in each round

### In-depth qualitative interviews with CPT staff

In-depth interviews were conducted with CPT staff from the implementation partners at three time points. Staff were selected purposively from a list of trained CPT staff to include a diversity of roles, levels of seniority and organisations. The same staff were interviewed during each round, except in cases where the selected individuals left the organisation, in which case another individual was selected from the sampling frame. Given the varying roles and levels of experience among the CPT staff, the interviews were unstructured and informed by a topic guide. Interviews were designed to explore expectations around the CPT approach, experiences with using the approach, challenges and mitigation strategies, trends in perceptions, and ideas for improving the CPT in the future. Interviews were conducted remotely in English and were audio recorded, and transcribed. The interviews duration was between 30–60 min. Interviews were conducted by researchers based in the United Kingdom, Lebanon and Zimbabwe.

### Phone-based interviews with populations

Repeat structured interviews were conducted over the phone with approximately 50 participants in each country between January and August 2021. Where possible, the same participants took part in all data collection rounds. See Supplementary Material 2 for detailed flow charts of participant recruitment in Zimbabwe and Lebanon. Participants were eligible to participate if they had been directly targeted by the implementation partner’s COVID-19 programmes and were over 18 years old. A list of eligible participants was shared by the implementation partners. In compiling these lists the partners asked community members if they were willing to share feedback about their programmes and recorded their phone number and basic socio-demographic data. Participants were selected purposively to represent a diversity of characteristics (e.g. gender, age, geographical location, and participation in different aspects of the programmes). For each round of data collection, each participant was called up to 3 times at various times during the day. When numbers were not answered, out of service, or when a participant had left the programmatic area, another participant was selected from the list. During the last round of data collection, new participants were not recruited because it would not be possible to compare their responses across multiple rounds of data collection. Interviews were conducted in local languages (Arabic, Ndebele or Shona) and included structured multiple-choice responses as well as open-ended qualitative questions. The interviews collected socio-demographic data, knowledge, behaviours and experiences related to COVID-19, and the acceptability, relevance, and effects of COVID-19 programming. Where possible, questions were drawn from existing surveys. The interview tool was piloted in both countries to arrive at appropriate translations and address any misunderstandings. The main content of the survey stayed the same across rounds of data collection, but some questions were refined, removed or added based on changing circumstances during the pandemic and learning from previous rounds of interviews. Interviews were conducted by research assistants in Lebanon and Zimbabwe. At the end of the interviews, research assistants gave participants some pre-prepared COVID-19 health messages, as well as information on local sources of support.

### Programmatic data

CPT reports were shared with the research team. These were developed by implementation partners and summarised patterns emerging from the CPT data and intended programmatic actions. Three learning workshops occurred in 2021– in February, May and at the end of data collection (June in Zimbabwe and September in Lebanon). The first two workshops were joint, with team members from Zimbabwe and Lebanon attending to allow for cross-country learning, whilst the last one was held in each country separately. A mix of CPT implementation staff and researchers were involved in all the workshops. On each occasion, the CPT implementation partners shared their findings from the CPT and reflected on progress. The research team shared initial patterns emerging from staff and population interviews. A set of actions to strengthen the CPT process for the remaining period were then decided upon. CPT staff interview guides were modified to track progress against these.

### Data collection process

Research teams consisted of two research assistants (one man, one woman) and one data analyst per country. Research staff were hired by Oxfam and Action Against Hunger and were embedded within these organisations but had no involvement in CPT implementation. Research staff at the London School of Hygiene and Tropical Medicine and the Lebanese American University provided remote support and training to these local teams. In both countries, data collection commenced after staff received the initial training on the CPT (August 2020) and concluded in September 2021. Initially, the same data collection timeline was to be used for Zimbabwe and Lebanon, but due to unforeseen events (e.g. the August 4 blast in Lebanon, COVID-19 lock-downs, power outages, etc.), research timelines had to be adjusted for each country accordingly.

### Data analysis

Audio recordings of in-depth interviews were transcribed. Structured interview responses were recorded on a computer in a standardised survey form in Survey CTO in Lebanon and in Kobo in Zimbabwe. For open-ended questions, research assistants typed short notes during the interview and then went back once the interview was completed to transcribe and translate the responses in full. Qualitative data were analysed thematically with the aid of NVivo (QSR International, Cambridge, MA), following an analysis process outlined by Braun and Clarke [32]. Coding was done deductively, informed by the study hypothesis, and used a ‘top down’ coding system based on the study and method objectives. The same codebooks were used in both countries to allow for comparisons. Emergent themes were identified across the entire dataset and refined. Coding and initial analysis was led by CH and EZ. Coding matrices and the initial analysis were shared with the rest of the researchers for verification. Quantitative data were descriptively summarised for each round of data collection to illustrate trends. Programme reports and meeting recordings were reviewed by researchers who made notes of relevant programmatic changes, process adaptions, and reflections.

### Ethics and consent

Ethical approval was obtained from the London School of Hygiene and Tropical Medicine ethics committee [22586], the Lebanese American University (LAU.SON.RD2.15/Dec/2020) and the Medical Research Council of Zimbabwe (MRCZ/A/2652). Participation in the study was voluntary, and all participants were only enrolled after receiving complete details of the study in their local language and providing verbal consent (for interviews with population members) or written consent (for staff interviews).

## Results

Below we present a study participants description and five key themes that emerged from the data. The first three themes are drawn from the interviews with CPT staff and the analysis of programmatic data and describe reflections on the CPT implementation process. The last two themes emerged from interviews with members of crisis-affected populations and reflect their opinions of the CPT informed COVID-19 response programmes.

### Study participants description

#### CPT implementation staff

Fifteen local and international CPT implementation staff were interviewed in the first round (7 in Zimbabwe and 6 in Lebanon and 2 staff in European organisational headquarters). 14 staff were interviewed in the following two rounds. These individuals represented all 5 implementation partners. Most staff had worked for these organisations for at least a year prior to the pandemic and many had several years of experience within the NGO sector. The selected staff had backgrounds in WASH engineering, public health promotion, protection, and programme management. This included staff who were involved in the CPT at a global headquarters level, at a country management level and those who were directly involved in collecting the information of people’s perceptions through their community-facing work.

#### Crisis-affected persons

A detailed description of the socio-demographic characteristics of the populations is provided in Supplementary Materials 3. In both countries, between 41 and 50 participants were involved in each round of data collection, approximately half of the participants were women. In Zimbabwe, most participants were 36 to 60 years of age (72%). The sample population in Lebanon was on average younger, with most participants being 18 to 34 years of age (60%). In both settings, the average household size was seven people. Most participants had some level of formal education (96% in Zimbabwe and 78% in Lebanon). In Zimbabwe, 54% of participants identified as self-employed (primarily engaged in agricultural work), whereas this was only 16% in Lebanon due to a larger proportion of the population reporting that they were unemployed (56%). By the last round of data collection, 37% of people in Zimbabwe and 11% of people in Lebanon reported that their employment status had changed during the pandemic. Household incomes also decreased during the pandemic, with this being the case for 51% of participants in both Zimbabwe and Lebanon. In Zimbabwe, 68% of participant households included a person who had a pre-existing medical condition which put them at risk of more severe COVID-19 symptoms, while in Lebanon, 46% of households had family members at higher risk. The majority of households in both countries also included people aged over 60.

#### Theme 1: Understanding what the CPT was and was not

Before implementation began in both countries, staff were trained on the CPT approach. The trainings took place over several online sessions due to restrictions on in-person meetings. Staff in both countries initially reported that the trainings were relatively comprehensive, although several admitted that there was a lot of information presented at once and it was difficult to “*grasp all the concepts*”. In the first round of interviews, many staff framed their understanding of the CPT as being similar to other data collection methods that they were familiar with, such as surveys or focus group discussions (FGDs):

*“I think it’s the same information we normally collect from FGDs that we will get from the CPT.”* (Round 1– Lebanon)

Others initially viewed the CPT as being similar to accountability mechanisms or post-distribution monitoring (a process done following the distribution of hygiene kits or other non-food items) that they had previously used. In subsequent rounds of interviews (after the first learning workshop and as staff had the opportunity to apply the CPT process), staff reflected that the training could have done more to differentiate the CPT process from these standard programmatic data collection methods:

*“In fact, the team misunderstood what [the CPT] is, they went out and surveyed people and we had to sort of like re-align that this is about you having conversations if you hear something interesting then you write it down*.” (Round 2– Zimbabwe)

Others felt that the training focused too heavily on training people on the data collection process but did not convey the full value of the approach for improving community engagement and programme design.

#### Theme 2: Strengthening key aspects of the CPT process

Given the need to urgently roll out the CPT process during the pandemic, staff reflected that the training and guidance they received disproportionally focused on learning how to use the mobile data collection tool and how to code the data appropriately. Substantially, less time was given to discussing the practicalities of perception collection and the subsequent stages in the process such as data analysis, data verification and triangulation, translating emergent patterns into programmatic adaptions, and ongoing monitoring of programmatic changes. In the absence of formal guidance describing each of these stages of the CPT process, staff learned from each other, sought support from headquarters advisors, and iteratively developed agreed ways of working.

##### Data collection

Due to some confusion about the approach initially, many staff actively solicited perceptions from community members they were working with. As time went on, however, staff reflected that about three quarters of the information on perceptions emerged naturally and without active prompting. For example, a community member might mention something about COVID-19 during a community event and the staff using the CPT would take a moment to ask them to elaborate on this and then seek their consent to note it down. The remaining proportion of perceptions were collected through active asking, which could involve the CPT staff member asking someone in the community about their opinions related to COVID-19 while doing prevention work.

Factors that hindered staff in documenting perceptions included having access to mobile devices that were incompatible with the data collection software, being able to only engage with communities via WhatsApp groups or phone hotlines during periods of lockdown in Lebanon, and the fact that staff had competing priorities and responsibilities:

*“[CPT] is not that very difficult to implement. But the issue is, you may not have ample time maybe to collect the perceptions, simply because you are committed to other work-related activities on daily basis.”* (Round 2– Zimbabwe)

Staff also identified weaknesses in the way data were collected, particularly in the early months of CPT use. In both countries, perceptions were typically documented by staff in English, even though perceptions were expressed by community members in their native language. Typically, this meant that perceptions became “*twisted or biased based on what the staff member thought”* and some degree of nuance was lost in the process of rapid translation. At the first learning workshop, staff decided to document perceptions in the language that they were expressed. While some participants reported struggling to make this shift, it did seem to improve data quality:

*“I think it has only made it better because we are capturing the real thing out there... I don’t have to crack my head to get it in English, it just comes out as it is. It’s more natural.”* (Round 2– Zimbabwe)

Staff also reflected that sometimes perceptions were summarised by staff (rather than written verbatim) or documented in a way that was hard for the data analysis team to understand. Others mentioned that perceptions were sometimes grouped together. This arose when staff would be in a group environment and might document the reflections of multiple individuals in one data entry. Again, once this was brought to the attention of staff, changes were made accordingly:

*“All the staff members improved, filling the CPT better, it stopped being like a kind of a survey, they started collecting more perceptions, more details. Like before the data cleaning, it used to take time, because they didn’t fill them properly”. *(Round 3– Lebanon)

##### Data analysis

In both countries, staff reported that the analysis of the CPT data were the most difficult part of the process; this was largely because staff were unclear on what had to be done at this stage and because most of the implementation team had limited previous experience with analysing qualitative data:

*“I needed more training on qualitative analysis to feel that I have the confidence to be able to for example grab this raw data and extract from it, to make of it qualitative analysis”* (Round 2– Lebanon)

Staff also felt that the process of analysing and summarising the CPT data via reports was challenging because it was time-consuming:

*“When we are preparing this big report and analysing the data and to share it…this needs time, more than expected.”* (Round 2– Lebanon)

Staff in both countries initially focused on quantitively summarising the data from the CPT. This included focusing on how many perceptions had been collected from different regions, the types of people that data had been collected from, and which theme was being most regularly recorded. While this was not the primary intention of the data analysis, it did highlight some limitations in programmatic reach. For example, in Lebanon, staff found few perceptions were being collected from people with disabilities, and therefore actively adjusted their programmes to reach these populations. In Zimbabwe, the team realised that the majority of perceptions were being gathered by certain members of staff and therefore tried to actively motivate and support others to collect more perceptions. However, teams realised that by solely focusing on numerical trends, they were overlooking the richness in the content of the data on perceptions:

*“I’ve been looking at some of the early dashboards…you’ve got all this information, but I don’t know how to use half of this, like do I care that most people are talking about government measures? Actually I care about what specific government measures and what people think of them….For me it has always come back to the most interesting data not being the numbers but actually the perceptions.”* (Round 1– Zimbabwe)

Following discussions in the learning workshops, both countries put strategies in place to strengthen the analysis of perception data and focus more on the qualitative content of perceptions. This typically involved adopting the following process for analysing CPT data: 1) cleaning the data and validating the coding, 2) visualising the data through graphs or Power BI dashboards, 3) filtering the perceptions based on the most commonly reported themes 4) discussing the specific perceptions within the key emergent themes and making plans for how to adjust programmes accordingly. Regular meetings involving all CPT implementation staff were key for facilitating this last stage in the analysis process. Towards the end of the CPT programme both country teams printed visualisations of trends in perception around key issues. These were then posted in public places or discussed in community events to explain programming choices, so that community members could understand better how their views were being used.

##### Regular meetings/discussions

CPT staff found the meetings beneficial because they brought together staff working across different sectors and this led to a more complete understanding of the data and a more diverse set of potential actions being considered:

*“The whole team will have discussions on topics and... on the action on how to respond or act depending on what we get from the data and perceptions. In general, as a meeting, its usefulness was very good, because there was a variety of people and a variety of sectors, there was WASH, protection teams, field staff, officers and the senior [staff], so there was a discussion of ideas from different perspectives."* (Round 3– Lebanon)

The meetings also proved useful for sharing tips on how to strengthen the quality of the data collection and to help the implementation teams to see the full value of the approach. However, these meetings were often dominated by senior staff and certain members of the CPT implementation team felt that they did not have the opportunity to fully participate in the discussions and interpretation of the data.

##### Triangulation and verification

In the first few months of the CPT use, little was done to validate or triangulate the patterns emerging in the CPT data as the focus was simply on strengthening processes for data collection and preliminary analysis. However, over time the teams in both countries started to cross-check the trends that were emerging through informal and formal processes. This included comparing the CPT trends to other available data, discussing findings with networks of stakeholders involved in COVID-19 response, or trying to verify experiences with community members. For example, in Lebanon, when the CPT perceptions started to indicate that people no longer felt it necessary to wear masks, they subsequently conducted observations in the informal settlements to assess whether this was true. In Zimbabwe, they compared CPT trends with epidemiological information (to understand whether shifts in perspectives reflected changing patterns in transmission) and with data that was being shared via the Cluster Coordination System or being described in the press. Staff in both countries used stakeholder coordination mechanisms as a way of disseminating findings and sense-checking these.

*“So what we do is kind of like identify the key perceptions that are coming out and then we kind of share them with [stakeholders] to say from your experience, whether it’s at a health centre, or people working with communities, you know does that trend resonate with what you are seeing or experiencing. So it’s the kind of informal engagement to verify what is coming out.”* (Round 2– Zimbabwe)

While not specified in the initial CPT steps, staff formalised this process of sharing learnings with stakeholders by developing regular CPT bulletins that highlighted key quantitative and qualitative patterns. Staff found these reports time consuming to produce but felt that they were key for helping to legitimise the process and motivate external actors to take action in response to emergent trends.

##### Programmatic adaptions and follow-up activities

Initially the process for moving from emergent CPT themes to programmatic adaptations was unclear for staff. For example, there was no guidance on whether it was necessary for a certain number of people to express a perception before it merited being discussed or programmes being adapted accordingly. Staff working within the Lebanon CPT implementation team had different views on the level of consensus needed within the perception data:

*“We used to wait for the data compilation to be able to take certain decisions and actions… we can’t generalize… I don’t want to jump into conclusions because of only one single perception… we should look at the numbers and percentages, they should be taken into consideration. We need to set a specific criterion. It should not be random.”* (Round 3– Lebanon).

Staff were also initially concerned about how programmatic ideas would be developed, funded and rolled out. Most assumed that it would be senior staff who analysed the CPT data and made the decisions about programmatic changes and were therefore surprised that the CPT actually led to a more consultative process of programmatic adaption:

*“What has been working well about the CPT is the sharing of information and bouncing ideas off each other to develop programme ideas”* (Round 3– Zimbabwe)

Staff also initially expressed concerns about the budgetary flexibility within their programmes:

“*I think the biggest aspect would be the budget you know your programming will be like already predetermined so in terms of the flexibility, you are limited there… We can’t move as broadly as we probably would like based on the perceptions.”* (Round 2– Zimbabwe)

However, in practice this was not a major challenge as the CPT tended to lead to ongoing programmatic tweaks rather than major changes to programme design:

*“We were not making a major shift or major transformation in the programs we have. [The CPT] highlights certain small gaps…and it highlighted certain things that we should be doing as a quick fix on those certain things.”* (Round 2– Lebanon)

The most common changes to programmes tended to be adaptations to messaging about COVID-19, with new information being developed based on the perceptions that emerged:

*“Basically those [CPT] perceptions have been used to craft key messages, the perceptions have guided us because we may end up doing the wrong thing if we do not have the perceptions, we may find our programmes would have misfired. So when using those perceptions, we exactly know what to target.”* (Round 3– Zimbabwe)

Perception trends emerging through the CPT were used to inform the creation of new videos or voice notes (e.g. content shared via social media and WhatsApp channels), communication materials (e.g. posters) or used to inform radio discussions. In Lebanon, the data indicated that many refugees struggled to access sufficient masks, prompting them to amend the product distributions they were doing. Refugees also expressed that they faced barriers in accessing the vaccines. This led the implementation team to support digital registration, provide transportation to clinics and ultimately seek further funding for vaccination promotion. Staff in Zimbabwe found it harder to make real-time changes to components of their work related to infrastructure provision, work in health centres, or within their complementary livelihoods initiatives, as these aspects of their work were less iterative and flexible. However, the CPT data were used to inform the development of subsequent grant proposals responding to the needs identified.

With time both implementation teams developed processes for moving from CPT insights to programmatic ideas more systematically. This involved using a table to track insights, programmatic recommendations and then monitor how changes were going:

*“There's a table. We record kind of a summary of the analysis and then we put the recommendation action and the status of what has been decided. It's great because we have a meeting every two weeks and use the same table and we add things. So we say, well, actually, that has been done, so we don't need to have this anymore. This is still relevant; this is what actually happened etc... So I think it's a nice way of monitoring as well in terms of linking the action and recommendation.”* (Round 3– Lebanon)

One unforeseen challenge was that it was not always possible for the implementing partners to directly address the trends emerging from the CPT data through their programming, because they related to factors outside their scope of work. This resulted in the implementing staff investing more time in disseminating the CPT findings externally and engaging in advocacy work with other actors (e.g. NGO partners or the government) in the hope of influencing change:

*“After collecting the perceptions, we realized that we could not effect some changes without the involvement of various government departments and that's when we had to go to them at a later stage and try to speak to them on what our findings were and how they could assist in making some changes.”* (Round 3– Zimbabwe)

#### Theme 3: The perceived value-add of the CPT to outbreak response programming

Staff in both countries were generally positive about the CPT approach from the outset, however, it took time for the teams to get used to the approach, fully embed it within their programming, and see its full value:

*“Initially we had taken [the CPT] on as a side activity. I think after some time, we've said, ‘Look, let's fully take it on board.’ Also when we took it as a side activity…some staff members did not to take time to really understand it and you know appreciate and be able to share; but gradually we got on top of the process.”* (Round 3– Zimbabwe)

By the third round of interviews, staff highlighted four key strengths of the CPT approach. Firstly, they felt it allowed them to do community engagement more systematically as it provided the evidence to support anecdotal observations:

*“I would say [the CPT] is making community engagement tangible… [The programmatic changes] could have happened without the CPT…but with the CPT, because it is a systematic and also because it is documented, then [the implementation teams] have the evidence that they can put on the table and say this is what people are telling us and this is how we should act.”* (Round 2– Lebanon)

Secondly, staff liked the “*organic*” nature of data collection which avoided “*top-down*” assumptions about what was driving behaviour. Specifically, the CPT process prompted staff to listen more and develop greater empathy and understanding of the experiences of members of the communities they were working with:

*“I'm overwhelmed by the CPT. I'm overwhelmed by the potential... It has achieved what I wanted to in the sense that it has made the team realise that part of our work is also to listen and to really kind of listen a little bit more than what we think we're listening. So in my view, even just achieving this is great.”* (Round 3– Lebanon)

Thirdly, staff reported that the CPT process was less time intensive and easier than they had initially expected and valued how quickly perceptions could be used to influence programming*.*

*“The CPT helps because we are collecting real-time data. During activities you can collect perceptions from the community members and then you can upload maybe let’s say by end of day, one can then analyse the perceptions given and by end of week, you can then respond to those perceptions in form of an intervention.”* (Round 3– Zimbabwe)

Lastly, participants explained that the CPT promoted integrated programming, bringing together staff and perspectives from different departments within their organisations (e.g. between WASH, health, protection, shelter and livelihoods teams) to ensure programming was more aligned and fully addressed emergent needs. The data from the CPT also helped the implementing organisations influence the work of other NGOs and government actors involved in the COVID-19 response.

Staff generally felt that the CPT had led to their programmes being considered as more acceptable and relevant by communities. However, given the multiple COVID-19 interventions being undertaken at the same time, most staff were unsure if the CPT had directly resulted in greater uptake of preventative behaviours during the pandemic. The teams in both countries were interested in continuing to use the CPT to inform their programming and expanding the process so that community members or government actors could be more involved in the data collection process.

The following two themes emerged from interviews with members of crisis-affected populations and reflect their opinions of the CPT informed COVID-19 response programmes.

#### Theme 4: Acceptability, relevance, and trust in COVID-19 prevention programmes

In both countries, most members of the crisis-affected population who were involved in the phone interviews reported being exposed to implementing partners’ programmes. Although participants were more able to recall programme components that involved the distribution of products or the provision of training rather than health promotion. There was an indication that some women in Lebanon may have been less exposed to the COVID-19 programming due to the modality of delivery:

*“The phone is not with me. It’s with the man. I don’t know. I don’t have a phone or a television.”* (Round 3– Lebanon)

When asked about COVID-19 information sources, participants in Lebanon relied quite heavily on NGOs for information and regarded this as the most trusted information source, with one participant explaining:

*“They [NGO’s] are the only ones standing by our side.”* (Round 5– Lebanon).

In Zimbabwe, the government and community health workers were more common and trusted sources of information throughout the pandemic than NGOs. However, in both countries trust in NGOs did appear to grow during the data collection period (see Supplementary Material 4 for detailed data related to this theme).

Participants in both countries felt that the programming done by the implementing organisations was generally beneficial and relevant:

*“The information and sessions are very beneficial and make us get information and raise our awareness, and the distributions are good especially that [preventative products] became expensive to buy and not always enough.”* (Round 4– Lebanon).

*"Yes, [the programmes] were relevant to our needs because I can see that our lives have improved from the way they were before".* (Round 4– Zimbabwe)

However, participants still felt that they needed more information or had needs related to COVID-19 that had not been fully met by NGO programming. For example, all participants in the round 4 interviews in Zimbabwe said they still had unmet information or resources needs, and in Lebanon, almost 36% indicated that they still needed more information or resources related to COVID-19. In terms of programme improvements, participants felt that the quality and quantity of the COVID-19 preventative products that were distributed could have been better. In Zimbabwe, some participants also raised concerns about the sustainability of the project and suggested expanding activities to other communities:

*“Maybe if they could increase the number of people in the community so that those that didn’t get the last time can also be helped. Because the things that they gave only help the recipient and their family and not the community at large."* (Round 2– Zimbabwe)

#### Theme 5: Self-reported changes in knowledge, beliefs and behaviour

Data from population interviews indicated that knowledge about COVID-19 symptoms was high in both countries at the start of data collection. For example, at the first round of data collection in Lebanon, 70% of people were able to list three or more symptoms of COVID-19, while this applied to 84% of participants in Zimbabwe (see Supplementary Material 5 for detailed data related to this theme). Knowledge about preventative behaviours was also high initially with 64% of participants in Lebanon being able to list four or more accurate preventative behaviours in round 1. In Zimbabwe, 42% were able to do the same in in round 1. At the last round of data collection, 97% of participants in Lebanon and 100% of participants in Zimbabwe believed that handwashing could reduce COVID-19 transmission. In Lebanon, 97% believed that masks were effective at reducing transmission, while 95% believed this in Zimbabwe. In both countries, 100% of participants believed physical distancing reduced transmission. If people developed COVID-19 symptoms, the most common actions participants said they would take were getting tested, going to a health centre, wearing masks, and trying to stay home more. In Zimbabwe, there were still several participants saying that if they got symptoms, they would focus on practicing a healthy lifestyle or using home remedies such as steaming and drinking herbal teas (*‘Zumbani’*).

*"My family and I have been taking water and heating it up if one feels like the situation is bad, then we all drink that hot water and steaming."* (Round 4– Zimbabwe)

In both countries, people reported dramatically increasing their handwashing behaviour during the first round of data collection (70% of people in Lebanon reported increasing their handwashing at the onset of the pandemic compared to 82% in Zimbabwe). Some people reportedly maintained this, but by the last round of data collection proportionally more people in Lebanon reported washing their hands less in the last month (42% ). In both countries, soap was available in the majority of households. In Lebanon, soap availability peaked during the round 2 interviews (89% of participants had soap available) and was lowest in round 5 (80% had soap available). In round 5, people were also less likely to report that they always used soap for handwashing. Soap access varied more in Zimbabwe but again peaked during the round 2 interviews with 96% of participants reporting having it in their household and again was lowest in round 5 with only 80% having access to soap. In Zimbabwe, it seems a larger proportion of people started using alcohol-based sanitiser during the pandemic, with it being used more frequently for handwashing in round 2 and 3 than soap. A large proportion of the population in Lebanon reported experiencing water insecurity (according to the 4 point HWISE scale [33]) with this being highest in rounds 1 (36%) and round 5 (63%). In the initial two rounds of data collection in Zimbabwe, 18% of people experienced water insecurity with this reducing in subsequent rounds.

During the initial rounds of data collection, there were lockdowns and people were encouraged to stay at home. In Lebanon, during round 1, 60% of people reported that they had not left their house in the last week. By round 2, this had fallen to 25% and after this, restrictions were relaxed. In Zimbabwe, 54% of people reported not leaving their home in the last week at the first round of data collection. Restrictions were subsequently relaxed, so this question was not asked again.

By the final round of data collection, only 2% of participants in Lebanon had received a COVID-19 vaccine, while 39% had received it in Zimbabwe. Participants were asked about their willingness to take vaccines from round 2 onwards and at this point 53% of Lebanese participants responded that they would be willing to take the vaccine, but this had fallen to 40% by the 5th round of data collection. In Zimbabwe 88% expressed willingness to take the vaccine in round 2 and this had risen to 98% in the final round.

Participants in both countries felt the programmes delivered by the implementing partners had played a role in their increased knowledge and led to behavioural change.

"*If COVID-19 hadn’t come, we wouldn’t have seen the likes of NAZ and Africa Ahead. We used to get water from the river because the boreholes were damaged. So, I think that because of COVID, maybe they saw that too many people might get sick and die because of dirty water and there would be too much poverty and they decided to help us. In our community we now… wash our hands with clean water so that we don’t get infected by COVID*"(Round 5–Zimbabwe)

*"[The programmes] affected us because we are not gathering, we are happy, we are cleaning and sanitizing because of the distributions, we always clean to protect ourselves from the virus, we wear masks they distribute, they always give us information about how to clean and stay at home and how to behave and this is very beneficial, we didn’t know these."* (Round 3–Lebanon)

People appreciated the combination of activities done by the implementing partners because they felt they would not have been able to apply their knowledge about preventative measures without the distribution of preventative products and improved access to infrastructure.

## Discussion

**Fig. 5:**
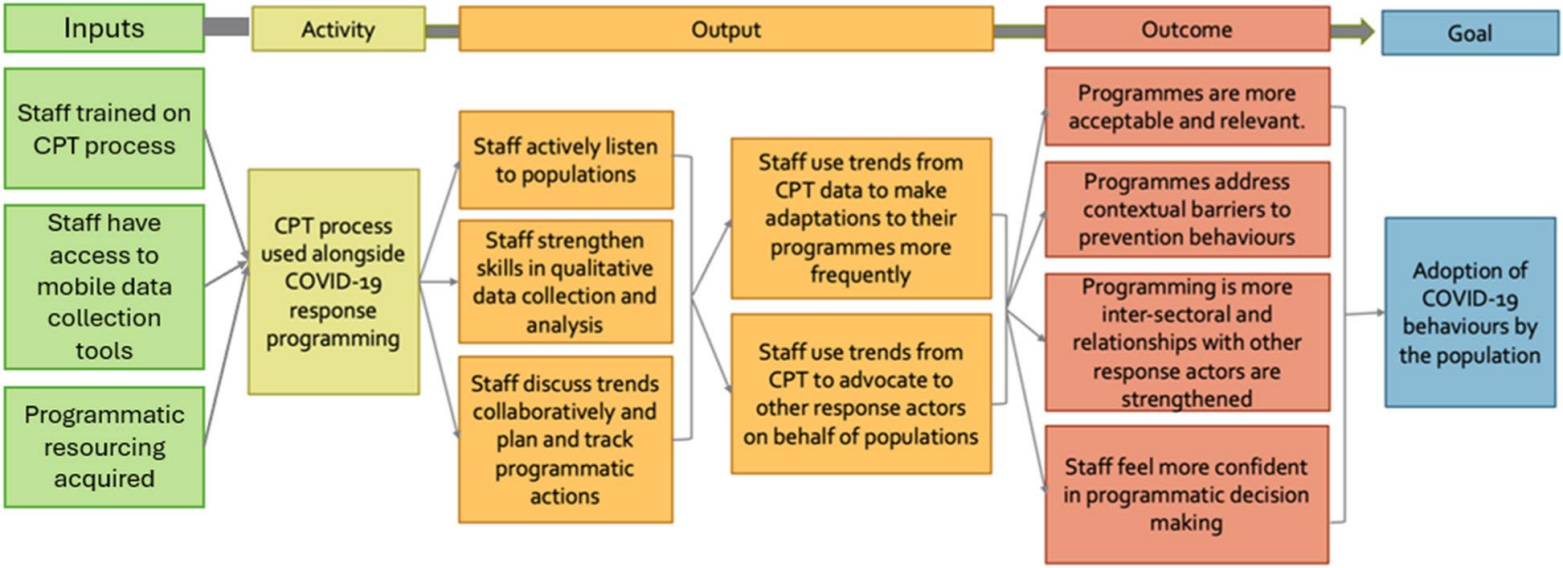
Revised theory of change for the community perception tracker based on findings

This phased, qualitative methods process evaluation was conducted to study the feasibility of using the CPT in COVID-19 response programmes and determine whether, in accordance with the hypothesised theory of change, this would result in programmes which were systematically adapted to the needs of community members so that they were ultimately more acceptable, relevant and effective. Overall, the CPT appears to be a promising tool for ensuring that community engagement is done more systematically throughout programme implementation and that community perspectives are actively used to improve programming. Our findings indicate that the core value of the CPT is to create a behavioural shift in the way humanitarian staff undertake programme implementation during outbreaks. While it took time to understand and implement the CPT process effectively, it was also well liked by staff and feasible to use within the scope of emergency programming. By comparing our findings to the hypothesised CPT theory of change, we found that change occurred through additional mechanisms, beyond what had been initially anticipated. Figure 5 shows a revised and expanded CPT theory of change which was developed based on our findings. At the output level it highlights the key aspects of the CPT that facilitated the behavioural shifts in programme design, such as active listening by staff, a strengthening of qualitative data collection and analysis skills and collaborative discussion of CPT data leading to the development of revised programme plans. This in turn meant that programmes were more frequently revised than they would ordinarily be the case. A functionality of the CPT, which was more important than initially anticipated, was that the data prompted staff to advocate to other actors on behalf of populations. At the outcome level the CPT had additional benefits to those initially hypothesised, including that staff felt more confident about programmatic decision-making and that programmes benefited from intersectoral inputs and stronger relationships with other response actors. It is plausible that these improvements to programme design and implementation had an impact on acceptability and relevance of the programmes, which may have an impact on programme effectiveness and the practice of COVID-19 preventative behaviours (as indicated by the self-reporting of behavioural adherence in our study). However additional research would be needed to verify behavioural practice and to more concretely attribute this to the use of CPT rather than other external factors.

Our findings are consistent with a similar study which assessed a community feedback mechanism that was utilised by the Red Cross during an Ebola outbreak in the DRC [34]. For example, just as we found that implementation teams initially focused on the quantitative aspects of CPT data collection, the authors in DRC found that the non-statistical approach to sampling community feedback, and its qualitative nature, meant that it was initially hard for the community feedback mechanism to be seen as a valid source of evidence to inform practice [34]. The authors also reported that it took time to generate buy-in around the novel approach from key stakeholders—yet this buy-in was essential for the findings to be able to influence outbreak programming [34]. Similarly, CPT staff reported actively investing in stakeholder engagement and cross-sector advocacy to ensure that findings beyond their operational remit could still affect change within the broader response. Finally, the authors of the DRC study also identified, like us, that the processing and analysis of large amounts of qualitative data are a specific technical skill set that is not always present within humanitarian organisations and which requires active capacity strengthening to support the analysis of community feedback [34]. Recent studies have emphasized the value of integrating social sciences into community engagement efforts in humanitarian programming and outbreak response, highlighting the need for systematic capacity development to support such integration in humanitarian programming [35–38].

Although the CPT was able to fulfil an important role in strengthening community engagement practices, it is likely that it may still need to be used alongside other tools to ensure community engagement is imbedded in all stages of humanitarian and outbreak response programming. By using the CPT, the implementing partners in Zimbabwe and Lebanon ensured programming met the ‘consult’ level of the Community Engagement Continuum [17, 18], with some aspects of programming exceeding this. For example, the CPT also facilitated better cooperation between response entities and, in cases where data validation processes were used, a more participatory flow of communication was established between the implementation actors and communities. The value of reaching this level of community engagement during the pandemic should not be understated given that response initiatives had to reduce in-person interactions and therefore often struggled to adapt programmes and engagement strategies [39] and because humanitarian staff were under a great deal of stress when designing and implementing programmes [40]. The CPT implementation partners are now exploring options for the CPT data collection and analysis process to involve crisis-affected populations and for community members to be involved in developing programme solutions. This would help the process further deepen its community engagement and move up the continuum.

If the CPT is to be used to inform programming in future outbreaks, there are several aspects of the process which could be strengthened. Staff were initially unclear on what the CPT was and the concrete steps required to implement it alongside programming. Therefore, it would be useful to indicate that training and support is a core part of the CPT implementation process. The development of a more robust training and support package should provide more guidance on qualitative analysis, team meetings, stakeholder engagement, and data sharing and validation.

Following on from this evaluation, the CPT has continued to be iteratively improved and rolled out in other settings. The Oxfam team were able to strengthen training processes and develop a more standardised CPT tool with guidance and feedback categorisations that work well across contexts. They are also exploring how AI might be able to support some of the qualitative analysis of perceptions and facilitate translation. These continued developments may facilitate uptake of the CPT. There may be some value in introducing CPT to governments in fragile or disaster prone regions so they are better able to respond if a crisis occurs.

### Reflections on the evaluation approach

Process evaluations are designed to understand ‘why’ and ‘how’ successful public health interventions create change [41, 42]. Standard process evaluations typically engage an external evaluator and present their findings at the conclusion of the intervention with the aim of informing future practice [43]. However due to the unprecedented nature of the pandemic and the need to adopt remote ways of conducting programmatic research, we adopted a novel phased evaluation process and one where the evaluation team were more actively engaged in iteratively improving practice. By breaking the evaluation into phases, that were punctuated with collaborative learning workshops, the CPT implementation team were able to reflect on the CPT process and develop plans for improving practice through a process of consensus building, which could then be followed up in the next phase by the evaluation team. This structured process of sharing learning was mutually beneficial and did not compromise our ability to objectively understand how change was occurring. Phased evaluation approaches may therefore be suitable for the evaluation of other interventions that take place during public health outbreaks or novel types of humanitarian crises.

Our evaluation also saw research staff being embedded within the implementation organisations, but not directly involved in the implementation. This afforded the researchers the ability to navigate both insider and outsider perspectives [44] and ‘walk alongside’ implementation teams [45] in order to more comprehensively understand how the CPT operated within the broader organisational dynamics and context. We envisioned that embedding research staff within these organisations would encourage skill-sharing related to data analysis, programmatic learning and research, practices that are often lacking within humanitarian organisations [46, 47]. However, we found that embedding research staff also created certain challenges. For example, the line between ‘research work’ and ‘implementation work’ became blurred, particularly when the research team possessed skills which were of use to the CPT implementation process (e.g. staff were sometimes asked for advice on the CPT data analysis). Declining such requests for support was challenging for the research teams given that they saw first-hand how stretched for time the implementing staff were during the pandemic. Another example of ‘role blurring’ arose during the phone interviews with populations. The research team were required to identify themselves as working for the implementing organisations, and as such we felt that there was also an ethical responsibility to provide COVID-19 information at the end of interviews, to correct potentially harmful misinformation. While these messages were standardised, this may have influenced later rounds of interviews with populations in terms of knowledge and practices. Where internal or embedded evaluators are used as part of future operational research, we would recommend being clear from the outset about roles and responsibilities and trying to pre-identify areas where roles may overlap.

### Limitations

We had initially proposed to include observational measures of COVID-19 prevention behaviours within the target sites; however, this was not possible. This was due to changes in programming in Lebanon, which meant that the handwashing facilities were provided at household levels rather than community levels, making it difficult to safely conduct observations during the pandemic. In Zimbabwe, there were delays in starting the observational data collection, which meant that it was no longer a useful method. As such our study is reliant on self-reported behaviour which other studies have shown to overestimate actual practice, particularly at a time where these behaviours were heavily promoted and therefore socially desirable [48–50].

During the interviews with population members, we emphasised that we wanted their honest views on programming and that any negative views expressed would not affect their access to programming or services. However, since they were all recipients of support of these programmes, many participants may still have provided socially desirable responses, focusing on the positive elements of programmes. Similarly, during interviews with CPT staff, participants may have been more likely to report positive aspects of their work as this would reflect better on them and their colleagues. These biases have been acknowledged as common challenges in the evaluation of humanitarian programming [51]. Additionally, interviews with staff were conducted in English, which could have limited the extent of feedback received.

Another limitation for the study was that interviews with the population were conducted remotely, via phone, due to the pandemic. This meant that only participants with a phone were eligible to participate. In Zimbabwe phone access is high (85% of the population) but this requirement may have excluded particularly marginalised groups. In Lebanon mobile access is lower (68%) and women are less likely to independently own phones which may have skewed responses [52]. Additionally, in order to obtain the number required of eligible participants, we had to rely on implementing partners providing us with phone numbers, which may have led to a bias in participants chosen.

Due to the nature of the pandemic our work was not accompanied by an impact evaluation and therefore we are unable to attribute any causality between CPT-related programme changes and the perceived acceptability of programmes or their impact on behaviour. Despite this we still feel the perspectives of implementation staff and affected populations are valuable for improving practice around community engagement.

## Conclusion

From the perspectives of implementation staff, the CPT was a feasible way of increasing community engagement throughout COVID-19 response programming. Staff valued the opportunity to strengthen their active listening skills and felt that the approach also led to more frequent and collaborative programme adaptation and greater cross-sectoral collaboration, both practices that are often lacking in during outbreaks [7, 53]. Although crisis-affected populations were generally positive about the programmes implemented and reported increased practices of COVID-19 preventative behaviours, our study was limited in its ability to verify actual behaviour and attribute change to the CPT process. Therefore, further research on the CPT is warranted. Despite these limitations, the phased process evaluation approach that we used could be replicated in other novel outbreaks where there is need for research and learning to immediately be shared with practitioners.

## Supplementary Information

Supplementary Material 1.

## Data Availability

The datasets used and/or analysed during the current study are available from the corresponding author on reasonable request.

## Abbreviations

ACF: Action Contre la Faim
CPT: Community Perception Tracker
DRC: Democratic Republic of Congo
FGDs: Focus group discussions
LMICs: Lower-and middle-income countries
NAZ: Nutrition Action Zimbabwe
NGO: Non-governmental organizations
TIDieR: Template for Intervention Description and Replication
WASH: Water, sanitation, and hygiene
