# Supplementary material for: Using the Community Perception Tracker (CPT) to inform COVID-19 response in Lebanon and Zimbabwe: a qualitative methods evaluation
Source: BMC Public Health. 2025 Aug 19;25:2850. doi: 10.1186/s12889-025-23755-4 (PMC12366037; doi:10.1186/s12889-025-23755-4)
Supplement: Supplementary file 1 — Supplementary Material 1. [file 12889_2025_23755_MOESM1_ESM.docx]

# Supplementary Material 1

## TIDierR Checklist describing the Community Perception Tracker approach as used in Lebanon and Zimbabwe during the COVID-19 pandemic.

| **Brief name** | |
| --- | --- |
| 1 | Community Perception Tracker (CPT) |
| **Why** | |
| 2 | The CPT enables community engagement to be systematically done alongside humanitarian or outbreak response programming. It is designed to encourage implementing programme staff to actively listen to communities, capture their perceptions, and analyse patterns in this perception data so that it can be used to support adaptive programme design that is relevant and acceptable to population needs. |
| **What** | |
| 3 | Materials: Detailed resources and information about the CPT process can be found at this website: <https://www.oxfamwash.org/en/communities/community-perception-tracker> |
| 4 | Procedure: The intended process of implementing is described below:  **Step 1 - Data collection:** While undertaking their planned programme work implementation staff are encouraged to actively listen to communities and note down any opinions they express about the outbreak (in this case COVID-19). This is done through the following actions:   1. The perception is noted down with the community member’s permission into a mobile-based data collection tool. 2. Additional information is noted about the date and location where data was collected, the characteristics of the person expressing the perception (e.g. sex, disability), how the person formed the perception (e.g. word of mouth from others, personal feeling, social media, etc), and what type of perception it was (e.g. a concern, a question, a belief or a practice). 3. Finally, the staff member categorises the perception against a set of pre-defined thematic categories (e.g. origin and existence of the disease, modes of transmission, preventative actions, impacts of the pandemic, vaccines, etc).   **Step 2 – Data Analysis:** Perception data is uploaded directly to a platform such as Survey CTO or Kobo so that data can be visualised through a semiautomated template on Power BI. This is intended to aid the first round of analysis. Additional data summaries and data analysis are conducted manually.  **Step 3 – Regular meetings and discussions based on the analysis of findings:** Programme staff, at all levels, meet regularly (e.g. bi-weekly) to review the perceptions data that are being collected.  **Step 4 - Triangulation with other actors and/or other teams within the organisation:** Implementation staff compare patterns and insights to other data sources, epidemiological data, or community and stakeholder perceptions to verify that emerging trends are valid.  **Step 5 - Adapting activities and influencing others:** Implementing actors collectively brainstorm appropriate programmatic adaptations. If findings relate to issues beyond their remit they identify and influence other actors who could enact change. Part of this process may involve developing reports which summarise quantitative and qualitative patterns in perceptions and can aid advocacy efforts.  **Step 6 - Follow up activities and monitoring:** Programmatic adaptations are implemented and monitored on an ongoing basis.  Over time, the CPT approach can be used to illustrate trends in perceptions across different locations, age, and gender groups. |
| **Who provided** | |
| 5 | The CPT approach can be used by any staff involved in the delivery of programmes to crisis or outbreak affected populations. In this case it was used by staff who were working on COVID-19 prevention programmes in Lebanon and Zimbabwe and who were employed by one of 5 implementing non-government organisations. These staff predominantly had backgrounds in water, sanitation and hygiene programming (either as engineers or doing public health promotion), protection programming or nutrition and livelihoods programming. Staff attended online training on the CPT approach before commencing use. |
| **How** | |
| 6 | The CPT was initially designed to be used alongside face-to-face response programming (such as during community events or household visits). However due to the nature of the COVID-19 pandemic it was also used as part of remote work (e.g. via phone hotlines or WhatsApp groups). Irrespective of the setting the approach is intended to record each individual perceptive separately and then look for trends across these. |
| **Where** | |
| 7 | During the COVID-19 pandemic the CPT approach was used in 12 low- or middle-income countries globally. In this study we report only the data from two countries: Lebanon and Zimbabwe. In both the CPT approach was used among populations that were at high-risk during the pandemic. In Lebanon this included Syrian refugees living in densely populated informal settlements and in Zimbabwe this included populations who were experiencing drought. |
| **When and How Much** | |
| 8 | The CPT approach was used by implementation on a routine basis from June 2020 in both countries. CPT use continues in both countries at the point of writing this paper. Team meetings to discuss the data typically happened on a bi-weekly basis and external reports were generated on a roughly quarterly basis. Programme adaptations and monitoring were undertake on a rolling basis. |
| **Tailoring** | |
| 9 | The CPT had only been piloted at a small scale prior to the pandemic. This evaluation aimed to document, among other things, whether it was implemented as intended in both countries and the ways that it varied from the intended delivery. Implementation staff were able to adapt the CPT process and adapt their programmes as they saw fit. This was typically done in discussion with managerial staff and in line with project proposals and budgets as agreed with donors. |
| **Modifications** | |
| 10 | Key modifications over the course of the CPT use included:   - More detailed guidance and processes for collecting perceptions, documenting these, categorising these and analysing these was developed. - The implementation staff developed standardised ways of organising the group discussions and formatting the reports so that these were as useful, and practice oriented as possible.   As these changes were made they were actively monitored and reflected on through the intervention. |
| **How well** | |
| 11 | Planned: Intervention fidelity was assessed by research staff by comparing practice, interview responses and reports to the intended process as initially defined by Oxfam. Preliminary findings and observations were shared back with implementation staff through three learning workshops. At the end of these sessions the implementation team agreed on ways of strengthening the CPT process and these were then monitored in the subsequent period by the research team. |
| 12 | Actual: Specific changes agreed to in the learning workshops were:  Data collection:   - To primarily collect perceptions naturalistically (i.e. without any active questioning) - To record perceptions verbatim and in the languages which they were expressed by the community member - To ensure that each recorded perception represented the views of one individual only.   Data analysis:   - To ensure the data analysis considered the qualitative content of perceptions as well as quantitative patterns across the themes   Reporting:   - To include within the report a table which described insights emerging from the CPT, proposed programme adaptation and a proposed plan for implementing this action and monitoring it. |

# Supplementary Material 2: Flow chart of participant recruitment in Zimbabwe and Lebanon for the phone-based interviews

Flow-chart for Zimbabwe

​

Initial recruitment (n=50)

Round 4 (n=41)

Included:

Verbal consent (n=5)

Round 2 (n=50)

Dropped out:

Unreachable (n=1)

Included:

Verbal consent (n=1)

Round 1 (n=50)

Dropped out:

Unreachable (n=5)

Round 3 (n=50)

Excluded:

Unreachable (n=3)

Unwilling to participate (n=2)

Out of country (n=1)

Sick (n=1)

No response (n=2)

Flow-chart for Lebanon

​

Initial recruitment (n=50)

Round 3 (n=47)

Round 4 (n=46)

Round 5 (n=45)

Round 2 (n=47)

Round 1 (n=50)

Excluded:

No response (n=1)

Number not in service (n=1)

Did not participate (n=1)

Included:

Verbal consent (n=3)

Excluded:

No response (n=2)

Did not participate (n=1)

Dropped out (n=3)

Included:

Verbal consent (n=3)

Excluded:

No response (n=3)

Did not participate (n=1)

Dropped out (n=3)

#

Excluded:

No response (n=1)

Number not in service (n=1)

Did not participate (n=3)

# Supplementary Material 3: Socio demographic characteristics of the populations in Zimbabwe and Lebanon.

| **Socio-demographic characteristics of the populations in Zimbabwe** | | | |
| --- | --- | --- | --- |
|  | **Total number of participants (N) = 50** | **Variable frequency/Total number of participants** |  |
| **Variable** | **n** | **%** | **Mean** |
| Sex | | | |
| Male | 24 | 48% | – |
| Female | 26 | 52% | – |
| Age | | | |
| 18-35 | 4 | 8% | – |
| 36-60 | 36 | 72% | – |
| >60 | 10 | 20% | – |
| Region | | | |
| Mwenezi | 24 | 48% | – |
| Chiredzi | 26 | 52% | – |
| Highest level of education | | | |
| No formal schooling | 2 | 4% | – |
| Some primary education or completed primary education | 19 | 38% | – |
| Some secondary school or completed secondary school | 26 | 52% | – |
| Some college or completed college/university | 3 | 6% | – |
| Occupation | | | |
| Unemployed (no work that earns money) | 20 | 40% | – |
| Self-employed (work in home: agriculture, potter, trader, etc) | 27 | 54% | – |
| Employed (work outside home, salaried: office, labourer, factory, etc) | 3 | 6% | – |
| Asset ownership (electricity, radio, television, computer, smart phone, refrigerator) | | | |
| 0 assets | 17 | 34% | – |
| 1-2 assets | 26 | 52% | – |
| 3-4 assets | 5 | 10% | – |
| 5-6 assets | 2 | 4% | – |
| Family members with pre-existing conditions that put them at risk of severe COVID-19 symptoms  Round 1 n=50  Round 4 n=41 | | | |
| No pre-existing health conditions | 16 (round 1)  16 (round 4) | 32% (round 1)  39.0% (round 4) | – |
| Family members with at least one pre-existing health condition | 34 (round 1)  25 (round 4) | 68% (round 1)  61.0% (round 4) | – |
| Average number of people living in household | – | – | 7.2 (round 1)  7.2 (round 4) |
| Average number of children <5 living in household | – | – | 1.2 (round 1)  1.3 (round 4) |
| Average number of people >60 living in household | – | – | 0.5 (round 1)  0.5 (round 4) |
| Change in occupation in last 6 months of data collection (n=41) | | | |
| Yes | 15 | 36.6% | – |
| No | 26 | 63.4% | – |
| Change of income in last month of data collection (n=41) | | | |
| Increased | 8 | 19.5% | – |
| Stayed the same | 12 | 29.3% | – |
| Decreased | 21 | 51.2% | – |

| **Socio-demographic characteristics of populations in Lebanon** | | | |
| --- | --- | --- | --- |
|  | **Total number of participants (N) = 50** | **Variable frequency/Total number of participants** |  |
| **Variable** | **n** | **%** | **Mean** |
| Sex | | | |
| Male | 25 | 50% | – |
| Female | 25 | 50% | – |
| Age | | | |
| 18-34 | 30 | 60% | – |
| 35-59 | 19 | 38% | – |
| ≥60 | 1 | 2% | – |
| Region | | | |
| Saiide | 25 | 50% | – |
| Boudai | 25 | 50 % | – |
| Highest level of education | | | |
| No formal schooling | 11 | 22% | – |
| Some primary education or completed primary education | 32 | 64% | – |
| Some secondary school or completed secondary school | 5 | 10% | – |
| Some college or completed college/university | 2 | 4% | – |
| Occupation | | | |
| Unemployed (no work that earns money) | 28 | 56% | – |
| Self-employed (work in home: agriculture, potter, trader, etc) | 8 | 16% | – |
| Employed (work outside home, salaried: office, labourer, factory, etc) | 14 | 28% | – |
| Asset ownership (electricity, radio, television, computer, smart phone, refrigerator) | | | |
| 0 assets | 1 | 2% | – |
| 1-2 assets | 17 | 34% | – |
| 3-4 assets | 31 | 62% | – |
| 5-6 assets | 1 | 2% | – |
| Family members with pre-existing conditions that put them at risk of severe COVID-19 symptoms  Round 1 n=50  Round 5 n=45 | | | |
| No pre-existing health conditions | 27 (round 1)  23 (round 5) | 54% (round 1)  51.1% (round 5) | – |
| Family members with at least one pre-existing health condition | 23 (round 1)  22 (round 5) | 46% (round 1)  48.9% (round 5) | – |
| Average number of people living in household | – | – | 7.3 (round 1)  7.8 (round 5) |
| Average number of children <5 living in household | – | – | 1.8 (round 1) |
| Average number of people >60 living in household | – | – | 0.3 (round 1)  0.2 (round 5) |
| Change in occupation in last 6 months of data collection (n=45) | | | |
| Yes | 5 | 11.1% | – |
| No | 40 | 88.8% | – |
| Change of income in last month of data collection (n=45) | | | |
| Increased | 8 | 17.7% | – |
| Stayed the same | 14 | 31.1% | – |
| Decreased | 23 | 51.1% | – |

*Missing data

# Supplementary Material 4: information sources and programme exposure of the populations in Zimbabwe and Lebanon.

| **Programming Data for Zimbabwe** | | | | | | | | |
| --- | --- | --- | --- | --- | --- | --- | --- | --- |
|  | **Round 1 (n=50)** | | **Round 2 (n=50)** | | **Round 3 (n=50)** | | **Round 4 (n=41)** | |
| **Variable** | **# per round** | **%** | **# per round** | **%** | **# per round** | **%** | **# per round** | **%** |
| Source of coronavirus information (multiple responses selected) | | | | | | | | |
| Mass media (television, radio, internet, newspaper/magazine, posters, information leaflet) | 25 | 21.7% | 21 | 19.3% | 17 | 14.7% | 24 | 27.3% |
| Social Media | 2 | 1.7% | 6 | 5.5% | 10 | 8.6% | 2 | 2.3% |
| Government, public health facility, community health worker | 68 | 59.1% | 54 | 49.5% | 54 | 46.6% | 33 | 37.5% |
| Community-level (Friends, spouse, other family, acquaintances/neighbours, church/mosque, community meeting/space, work) | 7 | 6.1% | 14 | 12.8% | 9 | 7.8% | 12 | 13.6% |
| NGOs (unspecified) | 13 | 11.3% | 14 | 12.8% | 26 | 22.4% | 17 | 19.3% |
| Most trusted source (single response selected) | | | | | | | | |
| Mass media (television, radio, internet, newspaper/magazine, posters, information leaflet) | 8 | 16% | 9 | 18% | 6 | 12% | 9 | 22% |
| Social Media | 0 | 0% | 0 | 0% | 1 | 2% | 0 | 0% |
| Government, public health facility, community health worker | 35 | 70% | 29 | 58% | 22 | 44% | 13 | 31.7% |
| Community-level (Friends, spouse, other family, acquaintances/neighbours, church/mosque, community meeting/space, work) | 0 | 0% | 1 | 2% | 2 | 4% | 2 | 4.9% |
| NGOs (unspecified) | 7 | 14% | 7 | 14% | 12 | 24% | 8 | 19.5% |
| Other | 0 | 0% | 4 | 8% | 7 | 14% | 9 | 22% |
| Have NGOs provided information or resources related to coronavirus? | | | | | | | | |
| Yes | 25 | 50% | 20 | 40% | 16 | 32% | 15 | 36.6% |
| No | 25 | 50% | 30 | 60% | 34 | 68% | 26 | 63.4% |
| Any other need for information/resources related to coronavirus in community that haven’t been adequately addressed | | | | | | | | |
| Yes | 48 | 96% | 46 | 92% | / | / | 41 | 100% |
| No | 2 | 4% | 4 | 8% | / | / | 0 | 0% |
| How much do you rely on word of mouth? | | | | | | | | |
| A lot | / | / | / | / | / | / | 22 | 53.7% |
| Sometimes | / | / | / | / | / | / | 10 | 24.3% |
| Not at all | / | / | / | / | / | / | 9 | 22.0% |

/ Data not collected

| **Programming Data for Lebanon** | | | | | | | | | | |
| --- | --- | --- | --- | --- | --- | --- | --- | --- | --- | --- |
|  | **Round 1**  **(n=50)** | | **Round 2**  **(n=47)** | | **Round 3**  **(n=47)** | | **Round 4**  **(n=46)** | | **Round 5**  **(n=45)** | |
| **Variable** | **# per round** | **%** | **# per round** | **%** | **# per round** | **%** | **# per round** | **%** | **# per round** | **%** |
| Source of coronavirus information (multiple responses selected) | | | | | | | | | | |
| Mass media (television, radio, internet, newspaper/magazine, posters, information leaflet) | 49 | 41.9% | 3 | 8.1% | 32 | 33% | 20 | 27.4% | 23 | 30.3% |
| Social Media | 6 | 5.1% | 3 | 8.1% | 14 | 14.4% | 13 | 17.8% | 9 | 11.8% |
| Government, public health facility, private health facility, community health worker | 6 | 5.1% | 1 | 2.7% | 4 | 4.1% | 3 | 4.1% | 1 | 1.3% |
| Community-level (Friends, spouse, other family, acquaintances/neighbours, church/mosque, community meeting/space, work, pharmacy) | 22 | 18.8% | 2 | 5.4% | 14 | 14.4% | 5 | 6.8% | 7 | 9.2% |
| NGO – Oxfam/Nabad | 27 | 23.1% | 23 | 62.2% | 29 | 30% | 28 | 38.3% | 29 | 38.2% |
| NGO – Other | 7 | 6.1% | 2 | 5.4% | 2 | 2.1% | 2 | 2.7% | 2 | 2.6% |
| No sources mentioned | 0 | 0% | 3 | 8.1% | 2 | 2.1% | 2 | 2.7% | 5 | 6.6% |
| Most trusted source (single response selected) | | | | | | | | | | |
| Mass media (television, radio, internet, newspaper/magazine, posters, information leaflet) | 14 | 28% | 3* | 9.7% | 5 | 10.6% | 5 | 10.9% | 2 | 4.4% |
| Social Media | 1 | 2% | 3* | 9.7% | 4 | 8.5% | 2 | 4.3% | 3 | 6.7% |
| Government, public health facility, community health worker | 2 | 4% | 1* | 3.2% | 1 | 2.1% | 2 | 4.3% | 1 | 2.2% |
| Community-level (Friends, spouse, other family, acquaintances/neighbours, church/mosque, community meeting/space, work) | 3 | 6% | 2* | 6.5% | 5 | 10.6% | 0 | 0% | 1 | 2.2% |
| NGO – Oxfam/Nabad | 21 | 42% | 17* | 54.8% | 25 | 53.2% | 27 | 58.7% | 25 | 55.6% |
| Other | 7 | 14% | 2* | 6.5% | 4 | 8.5% | 2 | 4.3% | 1 | 2.2% |
| No sources mentioned | 2 | 4% | 3* | 9.7% | 3 | 6.4% | 8 | 17.4% | 12 | 26.7% |
| Have NGOs provided information or resources related to coronavirus? | | | | | | | | | | |
| Yes | 47 | 94% | 44 | 93.6% | 42 | 89.4% | 45 | 97.8% | 44 | 97.8% |
| No | 3 | 6% | 3 | 6.4% | 5 | 10.6% | 1 | 2.2% | 1 | 2.2% |
| Any other need for information/resources related to coronavirus in community that haven’t been adequately addressed | | | | | | | | | | |
| Yes | 21 | 42% | 23 | 48.9% | 19 | 40.4% | 13 | 28.3% | 16 | 35.6% |
| No | 29 | 48% | 24 | 51.1% | 28 | 59.6% | 33 | 71.7% | 29 | 64.4% |
| Have NGOs provided information or resources related to coronavirus? | | | | | | | | | | |
| A lot | / | / | / | / | / | / | / | / | 6 | 13.3% |
| Sometimes | / | / | / | / | / | / | / | / | 23 | 51.1% |
| Not at all | / | / | / | / | / | / | / | / | 16 | 35.6% |

*Missing data

/ Data not collected

# Supplementary Material 5: Knowledge, perceptions and preventative behaviour data for populations in Zimbabwe and Lebanon

| **Knowledge and perceptions data for populations in Zimbabwe** | | | | | | | | | | | | | | | | |
| --- | --- | --- | --- | --- | --- | --- | --- | --- | --- | --- | --- | --- | --- | --- | --- | --- |
|  | | **Round 1**  **(n = 50)** | | | | **Round 2**  **(n = 50)** | | | **Round 3**  **(n = 50)** | | | | **Round 4**  **(n = 41)** | | | |
| **Variable** | | **#** | | **%** | | **#** | | **%** | **#** | | **%** | | **#** | | **%** | |
| Have you heard of disease COVID-19 | | | | | | | | | | | | | | | | |
| Yes | | 50 | | 100% | | / | | / | / | | / | | / | | / | |
| Who can become infected with coronavirus | | | | | | | | | | | | | | | | |
| # of participants who responded “Everybody” | | 32 | | 64% | | / | | / | / | | / | | / | | / | |
| # of participants who DID NOT respond “everybody” or “people who are not vaccinated against COVID-19” | | 28 | | 56% | | / | | / | / | | / | | / | | / | |
| Number of coronavirus prevention behaviours mentioned | | | | | | | | | | | | | | | | |
| Did not mention any behaviours | | 2 | | 4% | | / | | / | / | | / | | / | | / | |
| Mentioned 1-3 behaviours | | 27 | | 54% | | / | | / | / | | / | | / | | / | |
| Mentioned 4-6 behaviours | | 21 | | 42% | | / | | / | / | | / | | / | | / | |
| Mentioned more than 6 behaviours | | 0 | | 0% | | / | | / | / | | / | | / | | / | |
| What are the symptoms of coronavirus?  **(known symptoms: fever, headache, dry cough, diarrhoea, difficulty breathing, loss of smell, loss of taste, tiredness/fatigue, chills, sore throat, body aches, can be asymptomatic)** | | | | | | | | | | | | | | | | |
| Number of participants that were unable to identify any symptoms | | 1 | | 2% | | 1 | | 2% | 0 | | 0% | | 0 | | 0% | |
| Number of participants able to identify 1-2 symptoms | | 7 | | 14% | | 13 | | 26% | 10 | | 20% | | 3 | | 7.3% | |
| Number of participants able to identify 3 or more symptoms | | 42 | | 84% | | 36 | | 72% | 40 | | 80% | | 38 | | 92.7% | |
| Number of participants that mentioned **at least one** **incorrect** symptoms | | 18 | | 26% | | 22 | | 44% | 19 | | 38% | | 13 | | 31.7% | |
| Number of participants that mentioned **only** incorrect symptoms | | 0 | | 0% | | 2 | | 4% | 0 | | 0% | | 0 | | 0% | |
| Number of groups identified that are at high risk of getting really ill if they get coronavirus  **(high risk groups: pregnant women, elderly/over 60, people with HIV, people already sick/weak immune systems, people who are not vaccinated against covid-19, people who are diabetic, people with high blood pressure)** | | | | | | | | | | | | | | | | |
| Number of participants that were unable to identify any known high-risk groups | | 0 | | 0% | | 3 | | 6% | 2 | | 4% | | 0 | | 0% | |
| Number of participants that identified 1-2 known high-risk groups | | 36 | | 72% | | 31 | | 62% | 26 | | 52% | | 19 | | 46.3% | |
| Number of participants that identified 3 or more known high-risk groups | | 14 | | 26% | | 16 | | 32% | 22 | | 44% | | 22 | | 53.7% | |
| Personal chance of getting infected | | | | | | | | | | | | | | | | |
| No risk or Low risk | | 23 | | 46% | | 25 | | 50% | 25 | | 50% | | 8 | | 19.5% | |
| Medium risk | | 21 | | 42% | | 18 | | 36% | 21 | | 42% | | 13 | | 31.7% | |
| High risk | | 3 | | 6% | | 7 | | 14% | 4 | | 8% | | 20 | | 48.8% | |
| Already had coronavirus | | 1 | | 2% | | 0 | | 0% | 0 | | 0% | | 0 | | 0% | |
| Don’t know | | 2 | | 4% | | 0 | | 0% | 0 | | 0% | | 0 | | 0% | |
| Chance of someone in community getting coronavirus | | | | | | | | | | | | | | | | |
| No risk or Low risk | | 21 | | 42% | | 31 | | 62% | 20 | | 40% | | 3 | | 7.3% | |
| Medium risk | | 23 | | 46% | | 12 | | 24% | 25 | | 50% | | 30 | | 73.2% | |
| High risk | | 6 | | 12% | | 7 | | 14% | 5 | | 10% | | 8 | | 19.5% | |
| Level of concern if someone in household became infected | | | | | | | | | | | | | | | | |
| Not concerned | | 4 | | 8% | | 2 | | 4% | 2 | | 4% | | 2 | | 4.9% | |
| Mildly concerned | | 16 | | 32% | | 15 | | 30% | 20 | | 40% | | 10 | | 24.4% | |
| Very concerned | | 30 | | 60% | | 33 | | 66% | 28 | | 56% | | 29 | | 70.7% | |
| Does handwashing with soap/sanitizer reduce COVID-19 transmission | | | | | | | | | | | | | | | | |
| Yes | | / | | / | | / | | / | / | | / | | 41 | | 100% | |
| No | | / | | / | | / | | / | / | | / | | 0 | | 0% | |
| Does wearing masks reduce COVID-19 transmission | | | | | | | | | | | | | | | | |
| Yes | | / | | / | | / | | / | / | | / | | 39 | | 95.1% | |
| No | | / | | / | | / | | / | / | | / | | 2 | | 4.9% | |
| Does physical distancing reduce COVID-19 transmission | | | | | | | | | | | | | | | | |
| Yes | | / | | / | | / | | / | / | | / | | 41 | | 100% | |
| No | | / | | / | | / | | / | / | | / | | 0 | | 0% | |
| **Preventative behaviour data for populations in Zimbabwe** | | | | | | | | | | | | | | | | |
| Have you left your house in the last week | | | | | | | | | | | | | | | | |
| Yes | 23 | | 46% | | / | | / | | | / | | / | | / | | / |
| No | 27 | | 54% | | / | | / | | | / | | / | | / | | / |
| Most often wash hands at home (multiple responses selected in round 1; single response selected in round 4) | | | | | | | | | | | | | | | | |
| Fixed facility in own dwelling | 20 | | 40% | | / | | / | | | / | | / | | 4 | | 9.8% |
| Fixed facility in yard | 17 | | 34% | | / | | / | | | / | | / | | 26 | | 63.4% |
| Mobile object reported | 15 | | 30% | | / | | / | | | / | | / | | 4 | | 9.8% |
| Other | 14 | | 28% | | / | | / | | | / | | / | | 7 | | 17.1% |
| What is used to wash hands (multiple responses selected in round 1; single response selected in round 2, 3, 4) | | | | | | | | | | | | | | | | |
| Soap | 31 | | 62% | | 8* | | 16% | | | 14 | | 28% | | 21 | | 51.2% |
| Water-only | 8 | | 16% | | 5* | | 10% | | | 10 | | 20% | | 4 | | 9.8% |
| Ash | 7 | | 14% | | 2* | | 4% | | | 3 | | 6% | | 4 | | 9.8% |
| Alcohol-based hand rubs | 19 | | 38% | | 12* | | 24% | | | 20 | | 40% | | 12 | | 29.3% |
| Other | 2 | | 4% | | 1* | | 2% | | | 3 | | 6% | | 0 | | 0% |
| Currently have soap in household | | | | | | | | | | | | | | | | |
| Yes | 40 | | 80% | | 48 | | 96% | | | 47 | | 94% | | 33 | | 80.5% |
| No | 10 | | 20% | | 2 | | 4% | | | 3 | | 6% | | 8 | | 19.5% |
| In past 7 days, have you washed your hands with soap more often, less often or about same as prior to pandemic/last time we spoke | | | | | | | | | | | | | | | | |
| More | 41 | | 82% | | 24 | | 48% | | | 23 | | 46% | | 17 | | 41% |
| Less | 0 | | 0% | | 3 | | 6% | | | 4 | | 8% | | 1 | | 2% |
| Same | 9 | | 18% | | 23 | | 46% | | | 23 | | 46% | | 22 | | 54% |
| If you exhibit symptoms of coronavirus, what actions will you take (multiple responses selected) | | | | | | | | | | | | | | | | |
| Stay at home more | 3 | | 6% | | 1 | | 2% | | | 0 | | 0% | | 0 | | 0% |
| Stop attending social gatherings | 1 | | 2% | | 0 | | 0% | | | 0 | | 0% | | 0 | | 0% |
| Keep distance of at least 2m from others | 3 | | 6% | | 0 | | 0% | | | 1 | | 2% | | 0 | | 0% |
| Inform people of illness symptoms | 1 | | 2% | | 0 | | 0% | | | 1 | | 2% | | 0 | | 0% |
| Wash hands more frequently | 1 | | 2% | | 1 | | 2% | | | 2 | | 4% | | 0 | | 0% |
| Wear a mask | 2 | | 4% | | 0 | | 0% | | | 1 | | 2% | | 0 | | 0% |
| Go to health clinic | 28 | | 56% | | 14 | | 28% | | | 22 | | 44% | | 12 | | 29% |
| Go to be tested for coronavirus | 2 | | 4% | | 1 | | 2% | | | 1 | | 2% | | 12 | | 29% |
| Nothing | 0 | | 0% | | 0 | | 0% | | | 0 | | 0% | | 0 | | 0% |
| Practice healthy lifestyle | 0 | | 0% | | 0 | | 0% | | | 1 | | 2% | | 24 | | 59% |
| Get vaccinated against covid-19 | 0 | | 0% | | 0 | | 0% | | | 1 | | 2% | | 16 | | 39% |
| I will call the Covid-19 toll free number | 13 | | 26% | | 28 | | 56% | | | 28 | | 56% | | 0 | | 0% |
| Self-isolating | 12 | | 24% | | 13 | | 26% | | | 15 | | 30% | | 0 | | 0% |
| Call a health practitioner/Covid hotline | 12 | | 24% | | 9 | | 18% | | | 19 | | 38% | | 0 | | 0% |
| Other | 18 | | 36% | | 17 | | 34% | | | 12 | | 24% | | 7 | | 17% |
| If you were offered COVID-19 vaccine next month would you take it? | | | | | | | | | | | | | | | | |
| Yes | / | | / | | 43 | | 88% | | | 47 | | 94% | | 40 | | 98% |
| No | / | | / | | 5 | | 10% | | | 2 | | 4% | | 0 | | 0% |
| Not sure | / | | / | | 1 | | 2% | | | 1 | | 2% | | 1 | | 2% |
| Has anyone in your family received a COVID-19 vaccine? | | | | | | | | | | | | | | | | |
| Yes, they have received | / | | / | | / | | / | | | 9 | | 18% | | 16 | | 39% |
| Yes, family member received | / | | / | | / | | / | | | 9 | | 18% | | 8 | | 20% |
| No, they and family have not received | / | | / | | / | | / | | | 32 | | 64% | | 17 | | 41% |
| If vaccinated, would you continue to practice same behaviours to avoid getting coronavirus | | | | | | | | | | | | | | | | |
| Yes | / | | / | | / | | / | | | / | | / | | 41 | | 100% |
| No | / | | / | | / | | / | | | / | | / | | 0 | | 0% |

/ Data not collected

| **Knowledge and perceptions data for populations in Lebanon** | | | | | | | | | | | | | | | | | | | |
| --- | --- | --- | --- | --- | --- | --- | --- | --- | --- | --- | --- | --- | --- | --- | --- | --- | --- | --- | --- |
|  | | **Round 1**  **(n = 50)** | | | | **Round 2**  **(n = 47)** | | | | **Round 3**  **(n = 47)** | | | | **Round 4**  **(n = 46)** | | | **Round 5**  **(n = 45)** | | |
| **Variable** | | **#** | | **%** | | **#** | | **%** | | **#** | | **%** | | **#** | **%** | | **#** | **%** | |
| Have you heard of the disease COVID-19 | | | | | | | | | | | | | | | | | | | |
| Yes | | 50 | | 100% | | / | | / | | / | | / | | / | / | | / | / | |
| Who can become infected with coronavirus | | | | | | | | | | | | | | | | | | | |
| # of participants who responded “Everybody” | | 14 | | 28% | | 9 | | 19.2% | | 11 | | 23.4% | | 13 | 28.3% | | / | / | |
| # of participants who responded “People who are not vaccinated against COVID-19” | | / | | / | | 0 | | 0 | | 0 | | 0% | | 1 | 2.2% | | / | / | |
| # of participants who DID NOT respond “everybody” or “people who are not vaccinated against COVID-19” | | 36 | | 72% | | 38 | | 80.9% | | 36 | | 76.6% | | 32 | 69.6% | | / | / | |
| Number of coronavirus prevention behaviours mentioned | | | | | | | | | | | | | | | | | | | |
| Did not mention any behaviours | | 0 | | 0% | | 2 | | 4.3% | | 0 | | 0% | | 0 | 0% | | / | / | |
| Mentioned 1-3 behaviours | | 18 | | 36% | | 15 | | 31.9% | | 19 | | 40.4% | | 20 | 43.5% | | / | / | |
| Mentioned 4-6 behaviours | | 29 | | 58% | | 29 | | 61.7% | | 23 | | 48.9% | | 21 | 45.7% | | / | / | |
| Mentioned more than 6 behaviours | | 3 | | 6% | | 1 | | 2.1% | | 5 | | 10.6% | | 5 | 10.9% | | / | / | |
| What are the symptoms of coronavirus?  **(known symptoms: fever, headache, dry cough, diarrhoea, difficulty breathing, loss of smell, loss of taste, tiredness/fatigue, chills, sore throat, body aches, can be asymptomatic)** | | | | | | | | | | | | | | | | | | | |
| Number of participants that were unable to identify any symptoms | | 2 | | 4% | | 2 | | 4.3% | | 0 | | 0% | | 0 | 0% | | / | / | |
| Number of participants able to identify 1-2 symptoms | | 13 | | 26% | | 5 | | 10.6% | | 8 | | 17.0% | | 6 | 13.0% | | / | / | |
| Number of participants able to identify 3 or more symptoms | | 35 | | 70% | | 40 | | 85.1% | | 39 | | 83.0% | | 40 | 87.0% | | / | / | |
| Number of participants that mentioned **at least one** **incorrect** symptoms | | 22 | | 44% | | 22 | | 46.8% | | 16 | | 34.0% | | 18 | 39.1% | | / | / | |
| Number of participants that mentioned **only** incorrect symptoms | | 0 | | 0% | | 0 | | 0% | | 0 | | 0% | | 0 | 0% | | / | / | |
| Number of groups identified that are at high risk of getting really ill if they get coronavirus  **(high risk groups: pregnant women, elderly/over 60, people with HIV, people already sick/weak immune systems, people who are not vaccinated against covid-19, people who are diabetic, people with high blood pressure)** | | | | | | | | | | | | | | | | | | | |
| Number of participants that were unable to identify any known high-risk groups | | 9 | | 18% | | 5 | | 10.6% | | 7 | | 14.9% | | 5 | 10.9% | | 4 | 8.9% | |
| Number of participants that identified 1-2 known high-risk groups | | 41 | | 82% | | 41 | | 87.2% | | 40 | | 85.1% | | 37 | 80.4% | | 40 | 88.9% | |
| Number of participants that identified 3 or more known high-risk groups | | 0 | | 0 | | 1 | | 2.1% | | 0 | | 0% | | 4 | 8.7% | | 1 | 2.2% | |
| Personal chance of getting infected | | | | | | | | | | | | | | | | | | | |
| No risk or Low risk | | 28 | | 56% | | 21 | | 44.7% | | 19 | | 40.4% | | 30 | 65.2% | | 23 | 51.1% | |
| Medium risk | | 12 | | 24% | | 14 | | 29.8% | | 14 | | 29.8% | | 8 | 17.4% | | 13 | 28.9% | |
| High risk | | 5 | | 10% | | 6 | | 12.8% | | 6 | | 12.8% | | 5 | 10.9% | | 8 | 17.8% | |
| Already had coronavirus | | 0 | | 0 | | 3 | | 6.4% | | 5 | | 10.6% | | 3 | 6.5% | | 0 | 0% | |
| Don’t know | | 5 | | 10% | | 3 | | 6.4% | | 3 | | 6.4% | | 0 | 0% | | 1 | 2.2% | |
| Chance of someone in community getting coronavirus | | | | | | | | | | | | | | | | | | | |
| No risk or Low risk | | 14 | | 28% | | 11 | | 23.4% | | 13 | | 27.7% | | 18 | 39.1% | | 18 | 40% | |
| Medium risk | | 23 | | 46% | | 24 | | 51.1% | | 19 | | 40.4% | | 19 | 41.3% | | 19 | 42.2% | |
| High risk | | 7 | | 14% | | 10 | | 21.3% | | 15 | | 31.9% | | 7 | 15.2% | | 7 | 15.6% | |
| Don’t know | | 6 | | 12% | | 2 | | 4.3% | | 0 | | 0% | | 2 | 4.3% | | 1 | 2.2% | |
| Level of concern if someone in household became infected | | | | | | | | | | | | | | | | | | | |
| Not concerned | | 3 | | 6% | | 4 | | 8.5% | | 3 | | 6.4% | | 7 | 15.2% | | 6 | 13.3% | |
| Mildly concerned | | 8 | | 16% | | 8 | | 17.0% | | 12 | | 25.5% | | 6 | 13.0% | | 5 | 11.1% | |
| Very concerned | | 39 | | 78% | | 35 | | 74.5% | | 32 | | 68.0% | | 33 | 71.7% | | 34 | 75.6% | |
| Don’t know | | 0 | | 0% | | 0 | | 0% | | 0 | | 0% | | 0 | 0% | | 0 | 0% | |
| Does handwashing with soap/sanitizer reduce COVID-19 transmission | | | | | | | | | | | | | | | | | | | |
| Yes | | / | | / | | / | | / | | / | | / | | / | / | | 44 | 97.8% | |
| No | | / | | / | | / | | / | | / | | / | | / | / | | 1 | 2.2% | |
| Does wearing masks reduce COVID-19 transmission | | | | | | | | | | | | | | | | | | | |
| Yes | | / | | / | | / | | / | | / | | / | | / | / | | 44 | 97.8% | |
| No | | / | | / | | / | | / | | / | | / | | / | / | | 1 | 2.2% | |
| Does physical distancing reduce COVID-19 transmission | | | | | | | | | | | | | | | | | | | |
| Yes | | / | | / | | / | | / | | / | | / | | / | / | | 45 | 100% | |
| No | | / | | / | | / | | / | | / | | / | | / | / | | 0 | 0% | |
|  | |  | |  | |  | |  | |  | |  | |  |  | |  |  | |
| **Preventative behaviour data for populations in Lebanon** | | | | | | | | | | | | | | | | | | | |
| Have you left your house in the last week | | | | | | | | | | | | | | | | | | | |
| Yes | 20 | | 40% | | 35 | | 74.5% | | / | | / | | / | | | / | / | | / |
| No | 30 | | 60 % | | 12 | | 25.5% | | / | | / | | / | | | / | / | | / |
| Most often wash hands at home (single response selected) | | | | | | | | | | | | | | | | | | | |
| Fixed facility in own dwelling | 39 | | 78% | | 35 | | 74.5% | | / | | / | | / | | | / | / | | / |
| Fixed facility in yard | 4 | | 8% | | 4 | | 8.5% | | / | | / | | / | | | / | / | | / |
| Mobile object reported | 5 | | 10% | | 10 | | 21.3% | | / | | / | | / | | | / | / | | / |
| Public handwashing station | 2 | | 4% | | 2 | | 4.3% | | / | | / | | / | | | / | / | | / |
| What is used to wash hands (single response selected) | | | | | | | | | | | | | | | | | | | |
| Soap | 49 | | 98% | | 47 | | 100% | | 47 | | 100% | | 46 | | | 100% | 43 | | 95.6% |
| Water-only | 1 | | 2% | | 0 | | 0% | | 0 | | 0% | | 0 | | | 0% | 0 | | 0% |
| Ash | 0 | | 0% | | 0 | | 0% | | 0 | | 0% | | 0 | | | 0% | 0 | | 0% |
| Alcohol-based hand rubs | 0 | | 0% | | 0 | | 0% | | 0 | | 0% | | 0 | | | 0% | 2 | | 4.4% |
| Currently have soap in household | | | | | | | | | | | | | | | | | | | |
| Yes | 41 | | 82% | | 42 | | 89.4% | | 39 | | 83.0% | | 38 | | | 82.6% | 36 | | 80% |
| No | 9 | | 18% | | 5 | | 10.6% | | 7 | | 14.9% | | 8 | | | 17.4% | 9 | | 20% |
| In past 7 days, have you washed your hands with soap more often, less often or about same as prior to the pandemic/last time we spoke | | | | | | | | | | | | | | | | | | | |
| More | 35 | | 70% | | 17 | | 36.2% | | 18 | | 38.3% | | 9 | | | 19.6% | 7 | | 15.6% |
| Less | 1 | | 2% | | 6 | | 12.8% | | 11 | | 23.4% | | 10 | | | 21.7% | 19 | | 42.2% |
| Same | 14 | | 14% | | 24 | | 51.1% | | 18 | | 38.3% | | 27 | | | 58.7% | 19 | | 42.2% |
| If you exhibit symptoms of coronavirus, what actions will you take (multiple responses selected) | | | | | | | | | | | | | | | | | | | |
| Stay at home more | 12 | | 24% | | 6 | | 12.8% | | 11 | | 23.4% | | 6 | | | 13.0% | 9 | | 20% |
| Stop attending school or work | 4 | | 8% | | 1 | | 2.1% | | 3 | | 6.4% | | 0 | | | 0% | 0 | | 0% |
| Stop attending social gatherings | 3 | | 6% | | 0 | | 0% | | 0 | | 0% | | 2 | | | 4.3% | 0 | | 0% |
| Keep distance of at least 2m from others | 9 | | 18% | | 6 | | 12.8% | | 3 | | 6.4% | | 0 | | | 0% | 2 | | 4.4% |
| Inform people of illness symptoms | 2 | | 4% | | 2 | | 4.3% | | 2 | | 4.3% | | 1 | | | 2.2% | 1 | | 2.2% |
| Wash hands more frequently | 2 | | 4% | | 2 | | 4.3% | | 1 | | 2.1% | | 0 | | | 0% | 1 | | 2.2% |
| Wear a mask | 3 | | 6% | | 8 | | 17.0% | | 7 | | 14.9% | | 8 | | | 17.4% | 4 | | 8.9% |
| Go to health clinic | 30 | | 60% | | 24 | | 51.1% | | 19 | | 40.4% | | 17 | | | 37.0% | 17 | | 37.8% |
| Go to be tested for coronavirus | 15 | | 30% | | 21 | | 44.7% | | 18 | | 38.3% | | 18 | | | 39.1% | 19 | | 42.2% |
| Drink local alcohol | 0 | | 0% | | 0 | | 0% | | 0 | | 0% | | 0 | | | 0% | 0 | | 0% |
| Nothing | 0 | | 0% | | 0 | | 0% | | 0 | | 0% | | 1 | | | 2.2% | 0 | | 0% |
| Practice healthy lifestyle | 4 | | 8% | | 4 | | 8.5% | | 3 | | 6.4% | | 1 | | | 2.2% | 1 | | 2.2% |
| Get vaccinated against covid-19 | / | | / | | 0 | | 0% | | 2 | | 4.3% | | 1 | | | 2.2% | 1 | | 2.2% |
| Other | 15 | | 30% | | 7 | | 14.9% | | 1 | | 2.1% | | 1 | | | 2.2% | 2 | | 4.4% |
| If you were offered COVID-19 vaccine next month would you take it? | | | | | | | | | | | | | | | | | | | |
| Yes | / | | / | | 25 | | 53.2% | | 24 | | 51.1% | | 17 | | | 37.0% | 18 | | 40.9% |
| No | / | | / | | 11 | | 23.4% | | 14 | | 29.8% | | 18 | | | 39.1% | 15 | | 34.1% |
| Not sure | / | | / | | 11 | | 23.4% | | 9 | | 19.2% | | 11 | | | 23.9% | 11 | | 25% |
| Has anyone in your family received a COVID-19 vaccine? | | | | | | | | | | | | | | | | | | | |
| Yes, they have received | / | | / | | / | | / | | / | | / | | 0 | | | 0% | 1 | | 2.2% |
| Yes, family member received | / | | / | | / | | / | | / | | / | | 0 | | | 0% | 3 | | 6.7% |
| No, they and family have not received | / | | / | | / | | / | | / | | / | | 46 | | | 100% | 41 | | 91.1% |
| If vaccinated, would you continue to practice same behaviours to avoid getting coronavirus | | | | | | | | | | | | | | | | | | | |
| Yes | / | | / | | / | | / | | / | | / | | / | | | / | 40 | | 89% |
| No | / | | / | | / | | / | | / | | / | | / | | | / | 5 | | 11% |

/ Data not collected
